# From (Tool)Bench to Bedside: The Potential of Necroptosis Inhibitors

**DOI:** 10.1021/acs.jmedchem.2c01621

**Published:** 2023-02-13

**Authors:** Christopher R. Gardner, Katherine A. Davies, Ying Zhang, Martin Brzozowski, Peter E. Czabotar, James M. Murphy, Guillaume Lessene

**Affiliations:** † The Walter and Eliza Hall Institute of Medical Research, Parkville, VIC 3052, Australia; ‡ Department of Medical Biology, University of Melbourne, Parkville, VIC 3052, Australia; § Department of Pharmacology and Therapeutics, University of Melbourne, Parkville, VIC 3052, Australia

## Abstract

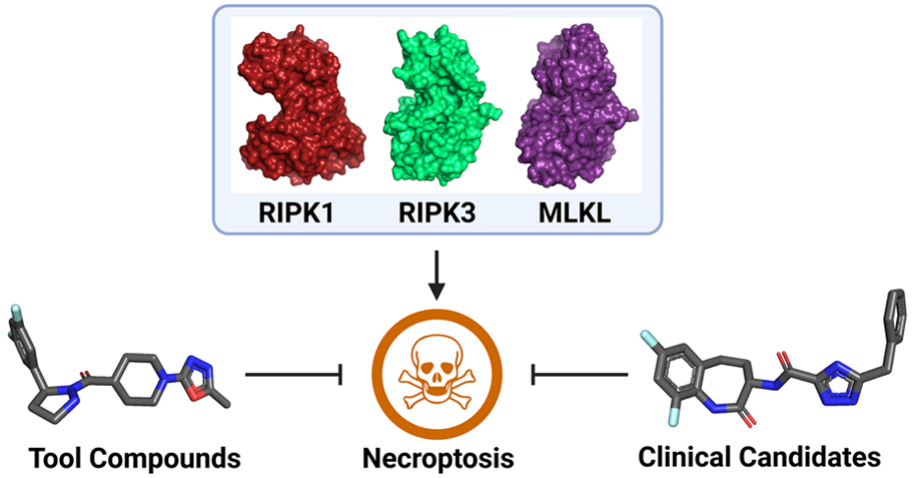

Necroptosis is a regulated caspase-independent form of necrotic cell death that results in an inflammatory phenotype. This process contributes profoundly to the pathophysiology of numerous neurodegenerative, cardiovascular, infectious, malignant, and inflammatory diseases. Receptor-interacting protein kinase 1 (RIPK1), RIPK3, and the mixed lineage kinase domain-like protein (MLKL) pseudokinase have been identified as the key components of necroptosis signaling and are the most promising targets for therapeutic intervention. Here, we review recent developments in the field of small-molecule inhibitors of necroptosis signaling, provide guidelines for their use as chemical probes to study necroptosis, and assess the therapeutic challenges and opportunities of such inhibitors in the treatment of a range of clinical indications.

## 1 Introduction

Cell death processes are fundamentally important to organism development and homeostasis. In addition to apoptosis, this century has seen the rise to prominence of several forms of regulated cell death, such as pyroptosis, ferroptosis, and necroptosis.^1^ Morphologically, necroptosis involves cellular swelling, the rupture of the plasma membrane, chromatin condensation, cellular lysis, and the loss of intracellular contents.^2^ Necroptosis can be induced by diverse stimuli including inflammatory markers such as tumor necrosis factor (TNF) receptor 1 (TNFR1), factor-associated suicide receptor (Fas), toll-like receptors 3/4 (TLR3/4),^3,4^ nucleotide binding and oligomerization domain (NOD)-like receptors (NLRs), interferon-α/β receptor subunit 1 (IFNAR1), and Z-DNA binding protein 1 (ZBP1).^5^ In the absence of pro-survival signals (via the nuclear factor κB (NF-κB) pathway) and caspase activity, a complex, termed the necrosome, is formed between receptor-interacting serine/threonine-protein kinase (RIPK) 1, RIPK3 and the mixed lineage kinase domain-like protein (MLKL) pseudokinase. Ultimately, RIPK3-mediated phosphorylation of MLKL leads to a conformational change,^6−8^ oligomerization,^9,10^ translocation to the plasma membrane, and membrane rupture,^9−12^ resulting in the release of damage-associated molecular patterns (DAMPs),^13^ inflammation, and organ injury.^14^

Animal models of disease and genetic experiments have demonstrated that necroptosis prominently contributes to a variety of disease pathologies, including neurodegenerative,^15−21^ cardiovascular,^22,23^ infectious,^24−31^ malignant,^32−36^ and inflammatory diseases.^35,37−47^ Furthermore, extensive studies have demonstrated that inhibiting the expression or activity of RIPK1, RIPK3, and MLKL is of therapeutic relevance. The identification of small-molecule inhibitors of necroptosis and their use as chemical biology tools has been essential to these discoveries, and their continued development underpins the efforts of researchers and clinicians in both understanding and treating necroptosis-related diseases. Throughout this Perspective, we aim to arm the reader with information vital to selecting appropriate tool compounds for inhibiting necroptosis *in vitro* and *in vivo*. For the purpose of this Perspective, *in vivo* animal models of necroptotic disease generally refers to rodent models easily accessible to most researchers. This distinction is important, as many inhibitors of necroptosis proteins display (in some cases) very high levels of specificity toward primate, in preference to rodent, orthologues of these proteins.

## 2 Molecular Functions of Necroptosis Regulator Proteins

**Figure 1:**
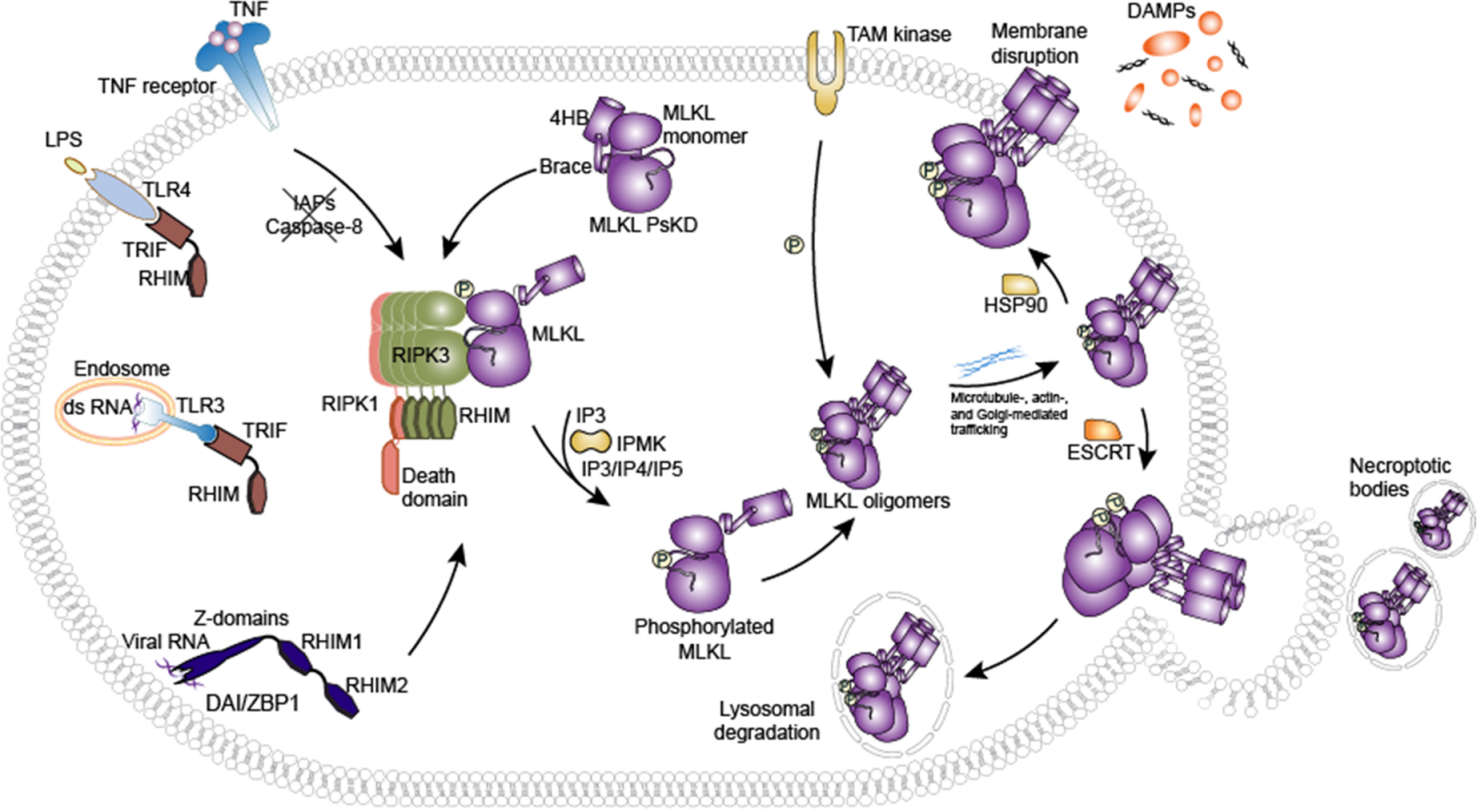
Signal transduction in necroptosis. Various pathways, including signals from death receptors, infection, inflammatory stimuli, and other cellular stresses, activate the necroptotic cell-death machinery.

Among the various necroptosis signaling pathways (Figure 1), the mechanisms downstream of TNFR stimulation have been best characterized. Upon the binding of TNF to TNFR1, cells are directed to either proinflammatory gene induction or the execution of cell death. Activated TNFR1 undergoes a conformational change and forms a complex, termed complex I, together with TNFR-associated death domain (TRADD), TNFR-associated factor 2 (TRAF2), RIPK1, cellular inhibitors of apoptosis 1 or 2 (cIAP1/2) and linear ubiquitin chain assembly complex (LUBAC).^48^ The ubiquitination of RIPK1 and other subunits in complex I by the E3 ubiquitin ligases cIAP1/2 and LUBAC leads to the subsequent recruitment of the transforming growth factor-β-activated kinase 1 (TAK1) complex and the inhibitor of nuclear factor κB kinase (IKK) complex to induce NF-κB and MAPK signaling.^49,50^ In scenarios where the activity of cIAP1/2 is compromised, TNF signaling is shifted to the induction of cell death via apoptosis through the formation of complex IIa (or ripoptosome) that comprises RIPK1, TRADD, Fas-associated protein with death domain (FADD), and caspase 8.^48,51^ When caspase activity is limited, cell death is redirected through necroptosis via the receptor interacting protein homotypic interaction motif (RHIM) domain-mediated association of RIPK3 to RIPK1 and formation of complex IIb in the cytoplasm.^24,52^ The RHIM domain-mediated homotypic interaction plays a crucial role in assembling subunit proteins into a higher-molecular-weight necrosome complex.^53^ Phosphorylated RIPK3 subsequently induces MLKL activation, which is represented by the phosphorylation of its pseudokinase domain, oligomerization, and translocation to the plasma membrane, where MLKL ultimately executes necroptotic cell death^6,9−11,54^ via an incompletely understood mechanism.^55,56^ Conversely, necroptosis originating from TLR3 or TLR4 stimulation proceeds through binding of toll/interleukin-1 receptor/resistance domain-containing adaptor-inducing interferon-β (TRIF), followed by the recruitment of RIPK1/RIPK3,^3,4^ while ZBP1, which detects cytosolic nucleotides, encodes a RHIM domain itself that allows for its direct interaction with RIPK1/RIPK3.^5^

As the core subunits of the necrosome (for further information on this concept, see Samson et al.^14^ and Horne et al.^57^), RIPK1, RIPK3, and MLKL are the most attractive targets of pharmacological inhibitors that aim to treat diseases involving necroptosis. RIPK1 is positioned at the intersection of cellular fates and plays a central role in directing the cell toward survival, or cell death via apoptosis or necroptosis. The role of RIPK1 in pro-survival signaling and the induction of inflammatory gene production is dependent on its scaffolding function. This role was demonstrated by studies in mice where RIPK1 knockout resulted in embryonic lethality,^58^ which was rescued by the expression of a kinase-dead RIPK1 mutant.^59,60^ Furthermore, cells expressing kinase-dead RIPK1 are capable of activating NF-κB signaling comparably to wild type cells.^58,61^ The kinase activity of RIPK1, which can induce autophosphorylation and the subsequently presumed conformational change that is crucial for caspase activation and necrosome formation, is required for the execution of several cell death modalities, including apoptosis, pyroptosis, and necroptosis.^24,48,62^

RIPK3 is required for necroptosis mainly in a RHIM domain- and kinase activity-dependent manner.^60,63−65^ RIPK3 has also been linked to other cellular pathways, including apoptosis, activation of the inflammasome, and induction of proinflammatory genes.^60,66,67^ It has been proposed that specific modifications in the kinase domain of RIPK3 can result in a pro-apoptotic conformation that leads to the formation of a death complex through RHIM domain-mediated interactions.^60,68,69^ The role of RIPK3 in promoting cytokine production has been reported in different diseases and genetic models;^67,70,71^ however, the underlying mechanisms are not fully understood.

MLKL is currently recognized as the terminal effector protein in necroptosis, although the mechanism of how MLKL is regulated has only been partially elucidated. Critically, the killer ability of MLKL is activated through the phosphorylation of its pseudokinase domain by RIPK3 and subsequent de-repression of its N-terminal executioner domain.^6,9^ Other mechanisms, such as those involving the ESCRT-III machinery, TAM kinases, inositol phosphate kinases, and trafficking via Golgi, microtubules, and actin, have also been reported to regulate MLKL in mediating necroptosis.^10,72−74^ The details of the post-translational modifications that control necroptosis signaling and other functions of the necroptotic proteins have been comprehensively reviewed elsewhere.^75^ The involvement of MLKL in other signaling pathways, such as inflammasome activation and autophagy, has been proposed but incompletely explored.^76^

In studies of necroptosis and its inhibition, cells are directed through this cell death pathway by the administration of a cocktail of reagents. The composition of this cocktail is dependent on the cell line being used (*i.e.*, whether or not the cell line is intrinsically deficient in caspase activation), the disease model being studied, and which effector (RIPK1, RIPK3, or MLKL) one is interested in studying or inhibiting. Such cocktails typically comprise one to three ingredients: a cell-death initiator, a second mitochondria-derived activator of caspases (SMAC) mimetic, and a caspase inhibitor. While necroptosis is best understood in the context of TNF activation, lipopolysaccharides (LPS), polyinosinic:polycytidylic acid (poly(I:C)), factor-associated suicide receptor (FasL), and interferon are also routinely used. SMAC mimetics are added to inhibit IAPs, thereby excluding RIPK1 from participating in prosurvival signaling and directing cellular fate toward death. Caspase inhibitors prevent cell death via apoptosis and redirect cell death via necroptosis. As with the choice of the cell death stimulus, it is imperative to carefully consider which caspase inhibitor is most appropriate for the system being studied. The most common caspase inhibitors used in studying necroptosis are zVAD.fmk (irreversible pan-caspase inhibitor),^77^ QVD-OPh (irreversible inhibitor of caspase 1, 3, 8, and 9),^78^ and IDN-6556 (emricasan, irreversible pan-caspase inhibitor).^79^ Aside from their differing potency against caspase 8 (the mediating caspase of necroptosis),^80−82^ it should also be noted that zVAD.fmk is capable of inhibiting other proteases at concentrations that are generally used in necroptosis assays.^83^

## 3 Role of Necroptosis in Disease Pathogenesis

Necroptosis has been implicated in the pathogenesis of many diseases spanning across broad disease areas, including inflammation,^35,37−47^ oncology,^32−36^ central nervous system (CNS)/neurodegeneration,^15−21^ and diabetes,^84^ as well as cardiovascular,^22,23^ kidney,^43,85,86^ liver,^40,87−89^ and infectious diseases.^24−31^ A range of experimental methods have been used to build evidence for the link between necroptotic proteins RIPK1/RIPK3/MLKL and disease pathogenesis. These methods include genetic experiments (knockout mice, shRNA or siRNA knockdown, and CRISPR Cas9 technology), pharmacological inhibition of RIPK1/RIPK3/MLKL proteins, protein/gene expression analysis of patient tissue samples, and *in vivo* animal disease models. A comprehensive analysis is outside the scope of this Perspective, and the reader is directed to key references and thorough reviews for specific disease classes. Additionally, an excellent review by Jouan-Lanhouet et al. summarizes tools for the *in vivo* detection of necroptosis in experimental disease models,^90^ and further information can be found in Samson et al.^91^ and Horne et al.^57^

It should be noted that although many studies linking necroptosis to a particular disease pathology have been reported, there is currently significant debate as to the validity of some of these studies (see examples in the context of liver disease,^92^ amyotrophic lateral sclerosis (ALS),^93,94^ colitis,^95^ nonalcoholic steatohepatitis (NASH),^96,97^ and pancreatic cancer^35^). A comprehensive investigation of several different mouse disease models by Newton et al., found that RIPK3 was dispensable in models of sepsis, colitis, pancreatitis, cerebral edema, and cerebral artery occlusion stroke.^98^ The authors hypothesized that RIPK1 and RIPK3 exhibit additional roles beyond the regulation of MLKL-dependent necroptosis, such as the promotion of apoptosis in certain contexts. Additionally, there is a possibility of RIPK3 loss leading to the suppression of pro-inflammatory cytokines and chemokines.^99^ Finally, information on murine models investigating the role of MLKL in diseases has been published.^100^ Taken together, these findings demonstrate the evolving nature of the field and should be carefully considered by researchers looking at developing novel therapeutics targeting necroptosis.

## 4 Development of Necroptosis Inhibitors

Since necroptosis was established as a regulated cell death pathway, the development of small-molecule inhibitors of necroptosis has played a critical role in delineating its underlying choreography and identifying the key proteins involved. Through a combination of classical screening approaches and cutting-edge technologies, we are now at a point where researchers have access to both tool compounds for *in vitro* and *in vivo* experiments (with significant species selectivity) and clinical candidates with the potential to offer breakthrough therapies for debilitating diseases.

### 4.1 Inhibitors of RIPK1

RIPK1 plays a decisive role in directing the cellular response toward either apoptosis or necroptosis. Several factors likely contributed to inhibitors of RIPK1 being the first inhibitors of necroptosis discovered. First, TNF is the most characterized and widely used stimulus to experimentally induce necroptosis, and this pathway operates through a RIPK1-dependent mechanism. Second, RIPK1 is a kinase, a class of proteins that is considered highly druggable due to the presence of a small-molecule binding site (ATP pocket) and an inhibitable catalytic function (phosphate transfer). Consequently, inhibitors of RIPK1 have been used to delineate the necroptosis pathway, identify the key effector proteins, and demonstrate the therapeutic potential of inhibiting necroptosis.

#### 4.1.1 Necrostatins

**Figure 2:**
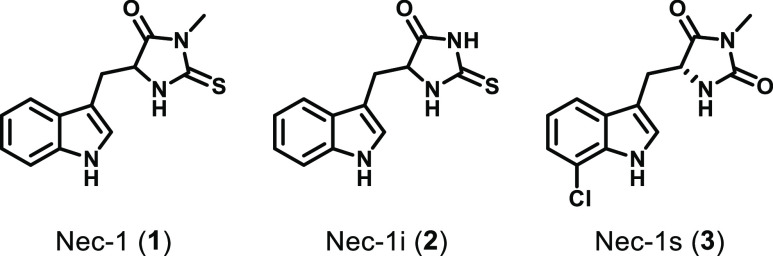
Chemical structures of the Nec-1 class of necrostatins.

Necrostatins are the prototypical inhibitors of necroptosis and have been indispensable for developing our current understanding of necroptosis biology. The necrostatins were identified in a phenotypic screen of a 15 000 compound library against human monocytic U937 cells treated with TNF and the pan-caspase inhibitor zVAD.fmk.^1^ This effort identified several diverse structural classes of compounds (necrostatin 1, 3, 4, 5, and 7), with the most used of these classes being the necrostatin 1 (Nec-1, **1**) family (Figure 2). As none of the other necrostatin classes have achieved the potency^101−105^ and subsequent widespread use of the Nec-1 class, they shall not be discussed further in this Perspective, other than to note that Nec-7 contains a known PAINS motif and its use should be avoided.^106^ Investigation of the structure–activity relationship (SAR) around the Nec-1 compound class (>200 analogues) in FADD-deficient Jurkat T cells revealed a limited scope for development, with most modifications being detrimental to activity.^107^ Of the analogues developed, the inactive Nec-1i (**2**; EC_50_ > 10 μM) and improved Nec-1s (**3**; EC_50_ = 50 nM) analogues are of note. Nec-1i was originally proposed as a suitable inactive control for experiments utilizing Nec-1; however, Nec-1i has also been shown to retain inhibitory activity against RIPK1 when used at higher concentrations *in vivo* (>10 μM), thereby limiting its utility.^108^ Furthermore, despite being remarkably selective for RIPK1 over other kinases,^109^ Nec-1 is only modestly potent (EC_50_ = 490 nM), unstable *in vivo* (*t*_1/2_ < 5 min in mouse liver microsomes (MLM)), and toxic at high concentrations.^107^ The use of Nec-1 and Nec-1i is further complicated by the observation that both are inhibitors of the immune regulator indoleamine 2,3-dioxygenase (IDO).^110^ This might confound the interpretation of experimental findings in inflammatory disease settings due to the high concentrations (typically tens of μM) at which Nec-1 is administered *in vivo*. In comparison to Nec-1, Nec-1s is not only more potent, more stable *in vivo* (*t*_1/2_ ∼ 60 min in MLM), and less toxic^107^ but is also not an inhibitor of IDO.^108^ However, the determination of the pharmacokinetic (PK) properties of Nec-1s (performed on the racemate) revealed its low exposure (AUC_8h_ = 0.27 μg h mL^–1^) and high clearance (61 mL min^–1^ kg^–1^).^107^ Despite these limitations on its *in vivo* use, Nec-1s remains an invaluable tool for cellular studies.

**Figure 3:**
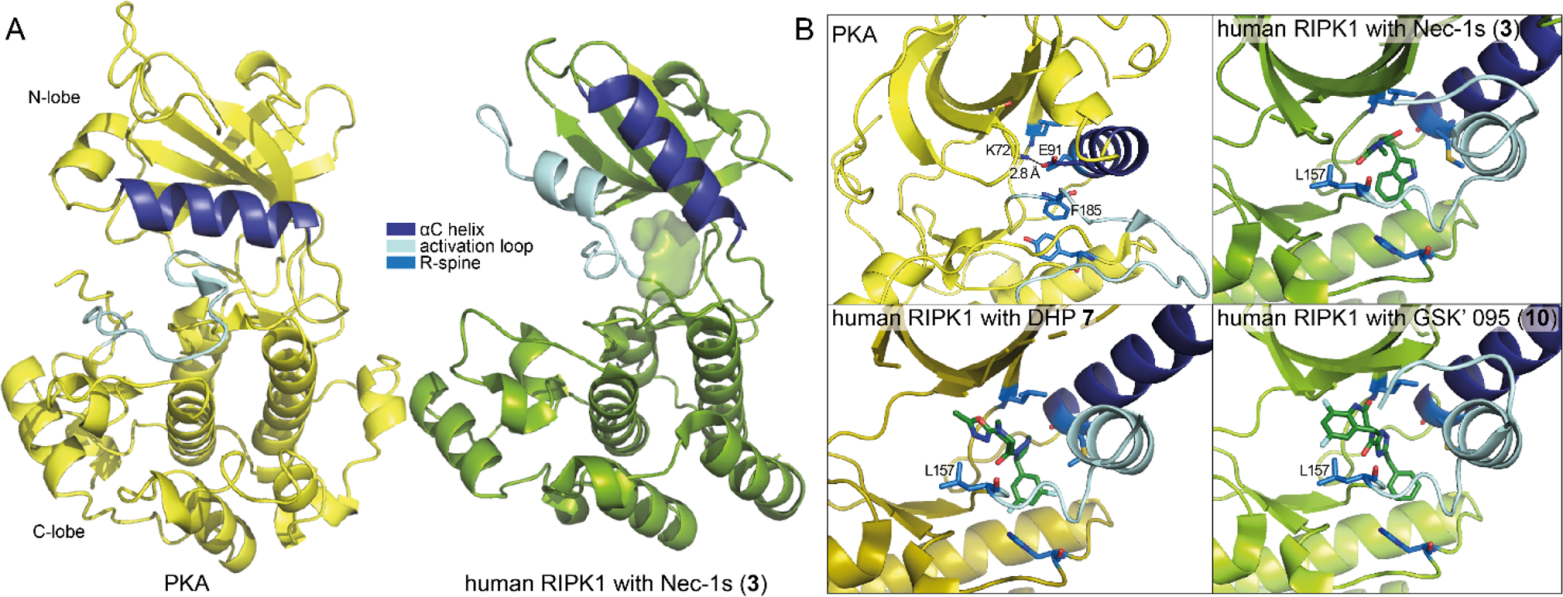
Selected crystal structures of RIPK1 with small-molecule inhibitors. (A) The apo human PKA in the active conformation (yellow; PDB 2CPK(113)) compared to human RIPK1 bound to Nec-1s, with the compound shown in surface view (green; PDB 4ITH(112)). The Nec-1s structure is representative of the inactive conformation of human RIPK1 bound to a type III inhibitor. (B) A comparison of the compound binding site of human RIPK1 and the equivalent site in active-conformation PKA, highlighting the regulatory spine rearrangement and the DFG motif F185 (PKA) or DLG L157 (human RIPK1) position. Compounds are shown in stick format.

Nec-1 and its derivatives were shown to inhibit RIPK1 in an ATP-competitive manner, thereby preventing its catalytic activity and rescuing cells from necroptosis.^111^ Through mutagenesis studies, it was established that autophosphorylation of the activation loop abrogated the inhibitory activity of Nec-1 analogues, suggesting a critical role for this segment in their mechanism of action, and that Nec-1 compounds bound to and stabilized an inactive DLG-out conformation of RIPK1.^111^ Subsequent disclosure of an X-ray crystal structure with Nec-1s bound to the kinase domain (residues 1–294, C34A, C127A, C233A, and C240A; PDB 4ITH) of RIPK1 revealed that the binding site was in fact a relatively hydrophobic allosteric pocket between the N-lobe and the C-lobe, designating Nec-1 compounds as type III allosteric inhibitors of RIPK1 (Figure 3).^112^ Binding of Nec-1s locks RIPK1 in an inactive conformation wherein the αC helix is displaced ∼40° relative to the catalytic subunit of protein kinase A (PKA; PDB 2CPK),^113^ with the vacated space being partially occupied by Nec-1s and the activation loop helix. Consequently, the salt bridge between E63 of the αC helix and K45 of the β3 strand (equivalent to E91–K72 in PKA, Figure 3B) that is required for the stabilization of ATP binding is also lost, with an E63–K45 separation of ∼15 Å. The inactive state of Nec-1s-bound RIPK1 is further exemplified through the characteristic “DLG-out” conformation of this motif, wherein D156 and S161 of the DLG motif interact with the Nec-1s inhibitor through two H-bonds. Finally, the hydrophobic regulatory (R)-spine typical of an active kinase conformation, which is formed by linear stacking of L78, M67, L157, and H136 in RIPK1, is disrupted, with M67 and L157 oriented away from the spine (Figure 3B). This allosteric binding mode accounts for the RIPK1 selectivity displayed by the Nec-1 class even against family members of the highest homology, namely, RIPK2 and RIPK3,^111^ due to the known benefits of type III inhibitors (*e.g.*, targeting less-conserved regions).^114^ The observed SAR of the Nec-1 class could also be rationalized through the hydrophobic nature of the pocket, the H-bond networks formed, and the proximity of Nec-1s to the residues within the narrow binding pocket. This X-ray structure represented a breakthrough in the field of RIPK1 inhibitor development, as it enabled pursuit of RIPK1 inhibitor structure-based design for the first time.^115^

#### 4.1.2 Dihydropyrazoles

**Figure 4:**
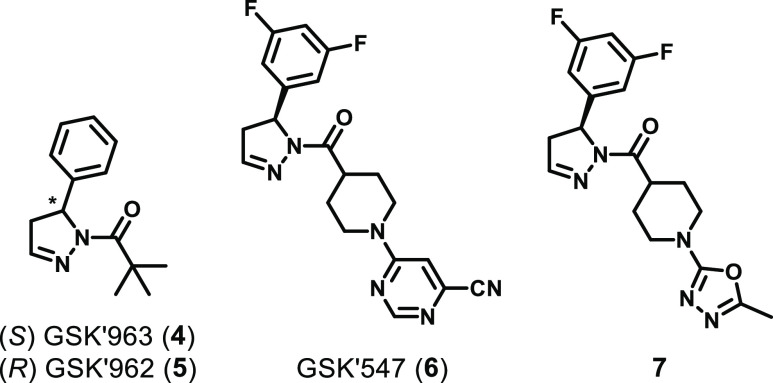
Chemical structures of the DHP class of inhibitors.

In 2015, GSK identified a dihydropyrazole (DHP) series of RIPK1 inhibitors by screening a 2 million compound library using an ADP-Glo enzymatic assay.^116^ Hybridization of the two screening hits and subsequent chiral resolution demonstrated the enantiospecific nature of their activity, with the (*S*)-enantiomer (GSK′963, **4**) being highly active in a fluorescence polarization (FP) binding assay (IC_50_ = 29 nM) and the (*R*)-enantiomer (GSK′962, **5**) being inactive at 10 μM (Figure 4).^116^ GSK′963 was also shown to be highly specific for RIPK1 when assessed against a panel of 339 kinases at 10 μM (*S*_(50)_ = 0.00, *S*_(20)_ = 0.01) and did not inhibit IDO.^116^ Furthermore, GSK′963 is efficacious in murine cells (IC_50_ = 1 and 3 nM against L929 and BMDM, respectively, both stimulated with TNF and zVAD.fmk) and is able to protect mice from TNF+zVAD.fmk-induced lethal shock (at 2 mg kg^–1^ and 0.2 mg kg^–1^ i.p.) *in vivo*.^116^ The exquisite kinase selectivity and nanomolar potency of GSK′963 make this inhibitor, coupled with its inactive isomer GSK′962, an excellent tool compound for evaluating necroptosis biology *in vitro*. However, due to its poor oral exposure (undetectable in rats) and short half-life (*t*_1/2_ = 3.5 and 20 min in rat and human microsomes, respectively), its use should be limited to acute models of necroptotic disease.^117^

A lead optimization campaign was later undertaken around these DHPs to improve the pharmacokinetic (PK) profile and generate *in vivo* tools. This led to the development of two improved inhibitors: **6** (GSK′547) and **7** (Figure 4).^117^ GSK′547 is a potential tool for *in vivo* studies in murine models of chronic disease due to improvements in potency (IC_50_ = 32 nM in L929 cells stimulated with TNF+QVD-OPh) and stability (*t*_1/2_ = 69 and 198 min in rat and human hepatocytes, respectively) and moderate PK properties in rats (i.v. and p.o.) relative to other analogues developed in the study.^117^ This was exemplified in an experimental autoimmune encephalomyelitis (EAE) mouse model of human multiple sclerosis and a Rd10 mouse model of human retinitis pigmentosa (RP). A delay in disease onset and reduced clinical severity were demonstrated in the EAE model, and protection of both retinal cell function and survival was observed in the Rd10 model.^117^

Importantly, an X-ray crystal structure of **7** bound to the RIPK1 kinase domain was obtained (PDB 6R5F) and showed that **7** occupies the allosteric region in the back of the ATP pocket (Figure 3B).^117^ Serendipitously, these inhibitors were found to occupy the same allosteric pocket as the Nec-1 series of compounds, with the overall structure of the RIPK1 protein being near indistinguishable from the Nec-1s-bound structure. The RIPK1–compound **7** structure enabled a rational explanation of the SAR observed for this chemical class. First, the ATP-competitive binding of **7** was supported by the orientation of the piperidine–oxadiazole portion toward the ATP pocket and the partial occupancy of the space where the ATP phosphates would be positioned. Second, the chair conformation of the piperidine complements the geometry of the narrow, hydrophobic binding pocket and orients the polar oxadiazole headgroup toward the solvent front.

#### 4.1.3 Benzoxazepinones

**Figure 5:**
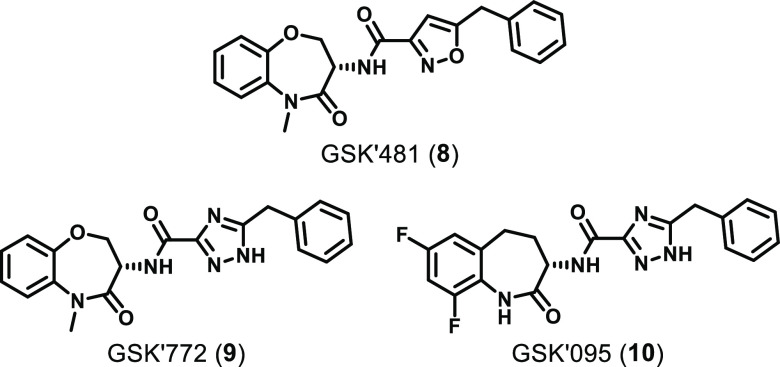
Chemical structures of the benzoxazepinones developed by GSK.

The benzoxazepinone series of RIPK1 inhibitors (Figure 5) was identified through a DNA-encoded library (DEL) screen against the RIPK1 kinase domain (residues 1–375).^118^ An optimization campaign yielded the lead benzoxazepinone **8** (GSK′481), which was active in RIPK1 FP and ADP-Glo biochemical assays (IC_50_ = 10 and 1.6 nM, respectively), and a U937 TNF+zVAD.fmk cellular assay (IC_50_ = 10 nM).^118^ GSK′481 also demonstrated complete kinome selectivity against two separate kinase panels (318 and 456 kinases, respectively) at 10 μM.^118^ However, the pronounced species selectivity of GSK′481 toward primate RIPK1 over rodent RIPK1 results in significantly reduced biochemical potency (200- and 329-fold higher IC_50_ values for mouse and rat, respectively, vs that for humans in the RIPK1 FP assay), and cellular efficacy in mouse L929 cells (IC_50_ = 3.2 μM),^118^ rendering GSK′481 useful only as an *in vitro* tool in human cells. This is further reinforced by its pharmacokinetic (PK) profile in rats, where GSK′481 exhibits low oral exposure (AUC_0–∞_ = 0.38 μg h mL^–1^ at 2 mg kg^–1^), high clearance (69 mL min^–1^ kg^–1^), and a high volume of distribution (8.5 L kg^–1^).^119^

Extensive SAR exploration around the benzoxazepinone scaffold culminated in the two clinical candidates **9** (GSK′772) and **10** (GSK′095) (Figure 5).^119,120^ Compared to GSK′481, GSK′772 exhibited improved efficacy in the ADP-Glo biochemical and U937 cellular assays (IC_50_ = 1.0 and 6.3 nM, respectively) while maintaining complete kinase selectivity toward RIPK1.^120^ When tested for activity in a Tox panel, GSK′772 displayed a generally clean profile with only weak activity against CYP2C9 (IC_50_ = 25 μM), weak inhibition of hERG in HEK293 cells (estimated IC_50_ of 195 μM), and weak activation of the human Pregnane X receptor (hPXR, EC_50_ = 13 μM).^120^ GSK′772 also displayed pronounced species selectivity toward primate RIPK1 (156- and 125-fold vs mouse and rat, respectively), resulting in significantly reduced cellular efficacy in murine L929 cells treated with TNF and the pan-caspase inhibitor QVD-OPh (IC_50_ = 3.2 μM).^120^ However, GSK′772 showed favorable PK properties in rats and monkeys, with moderate exposure (AUC_0–∞_ = 2.3 μg·h mL^–1^ at 2.1 mg/kg and 2.9 μg·h mL^–1^ at 1.9 mg/kg, respectively), low clearance (17 and 10 mL min^–1^ kg^–1^, respectively), a low volume of distribution (2.7 and 2.2 L/kg, respectively), good stability (*t*_1/2_ = 3.9 and 6.5 h, respectively), and ample safety windows over a one month safety assessment.^120^ Furthermore, GSK′772 was efficacious at inhibiting necroptosis (stimulated with TNF, a SMAC mimetic, and either QVD-OPh or zVAD.fmk) in primary human neutrophils, human whole blood, ulcerative colitis explant tissue, and TNF- or TNF and zVAD.fmk-induced mouse models of lethal shock despite the significant reduction in activity observed upon testing in murine cells vs human cells.^120^ GSK′772 has also completed phase IIa trials in patients with moderate to severe rheumatoid arthritis (NCT02858492),^121^ ulcerative colitis (NCT02903966),^122^ or plaque-type psoriasis (NCT02776033).^123^ The results of these trials suggest that GSK′772 is not suitable as a monotherapy for rheumatoid arthritis or ulcerative colitis.^121,122^ However, the outcomes of the trial in mild-to-moderate psoriasis suggest that inhibition of RIPK1 might have an impact on disease and that further studies are warranted with higher doses of GSK′772 and in patients with more active disease.^123^

GSK′095 similarly showed efficacy in ADP-Glo biochemical and U937 cellular assays (IC_50_ = 6.3 and 10.0 nM, respectively) while maintaining complete kinase selectivity toward RIPK1.^119^ GSK′095 also demonstrated high species selectivity toward primate RIPK1 and a good PK profile across rat, dog, and monkey models, with low clearance (27, 9.8, and 6.4 mL min^–1^ kg^–1^, respectively), a moderate volume of distribution (1.8. 1.1, and 1.8 L/kg, respectively), short-to-moderate terminal half-life (2.2, 1.7, and 4.2 h, respectively), and good oral bioavailability (84, 77 and 88%, respectively).^119^ Furthermore, GSK′095 was efficacious at inhibiting necroptosis (stimulated with TNF, a SMAC mimetic, and either QVD-OPh or zVAD.fmk) in human whole blood and in *ex vivo* tumor cultures of patient-derived organotypic spheroids (PDOTS) from freshly resected pancreatic, adenocarcinoma, colorectal, breast, and gastric cancer patients.^119^ GSK′095 recently completed a phase I clinical trial for pancreatic adenocarcinoma and other advanced solid tumors (NCT03681951).^119^

X-ray crystal structures of GSK′481, GSK′772, and GSK′095 bound to the RIPK1 kinase domain (residues 1–294, C34A, C127A, C233A, and C240A; PDBs 5HX6, 5TX5 and 6RLN, respectively) revealed that the benzoxazepinones are another class of type III inhibitors, which also bind to the same allosteric pocket as the necrostatins and DHPs.^118−120^ In fact, these crystal structures were obtained by first reproducing the published cocrystal structure of Nec-4 bound to this RIPK1 construct and then displacing Nec-4 with GSK′772 or GSK′095.^112^ Overall, the structure of RIPK1 is highly similar between the benzoxazepinones and other type III inhibitor-bound structures (Figure 3B). The benzoxazepinones all form H-bond interactions with the backbone residues of the DLG motif, which is in a classical “DFG out” conformation. In each structure, the benzoxazepinone moiety resides in a tight pocket formed by two β-strands defined by L90–V91–M92 and I43–M44–K45, with the benzyl group occupying an allosteric lipophilic pocket at the back of the ATP binding site. The benzoxazepinone ring also overlaps with space that would be occupied by the ATP α-phosphate, making these compounds ATP-competitive. Furthermore, the benzyl group of the compound takes the place of L157 from the DLG motif and forms an interaction with H136 of the HRD motif, potentially contributing to the stability of the inhibitor-bound conformation.^118−120^ Our understanding of the strong primate specificity of many of the type III RIPK1 inhibitors is hampered by the lack of any published nonhuman RIPK1 structures. However, through identification of the binding site and subsequent sequence alignments of primate and nonprimate RIPK1, it was observed that the sequence differences in the activation loop, the C-helix, and the glycine-rich loop might result in nonprimate RIPK1 having less flexibility to undergo the significant conformational reordering required to bind the benzoxazepinones.^118^ Mutagenesis studies then confirmed the activation loop as the key region in murine RIPK1 that is responsible for the primate species selectivity of the benzoxazepinones.^118^

**Figure 6:**
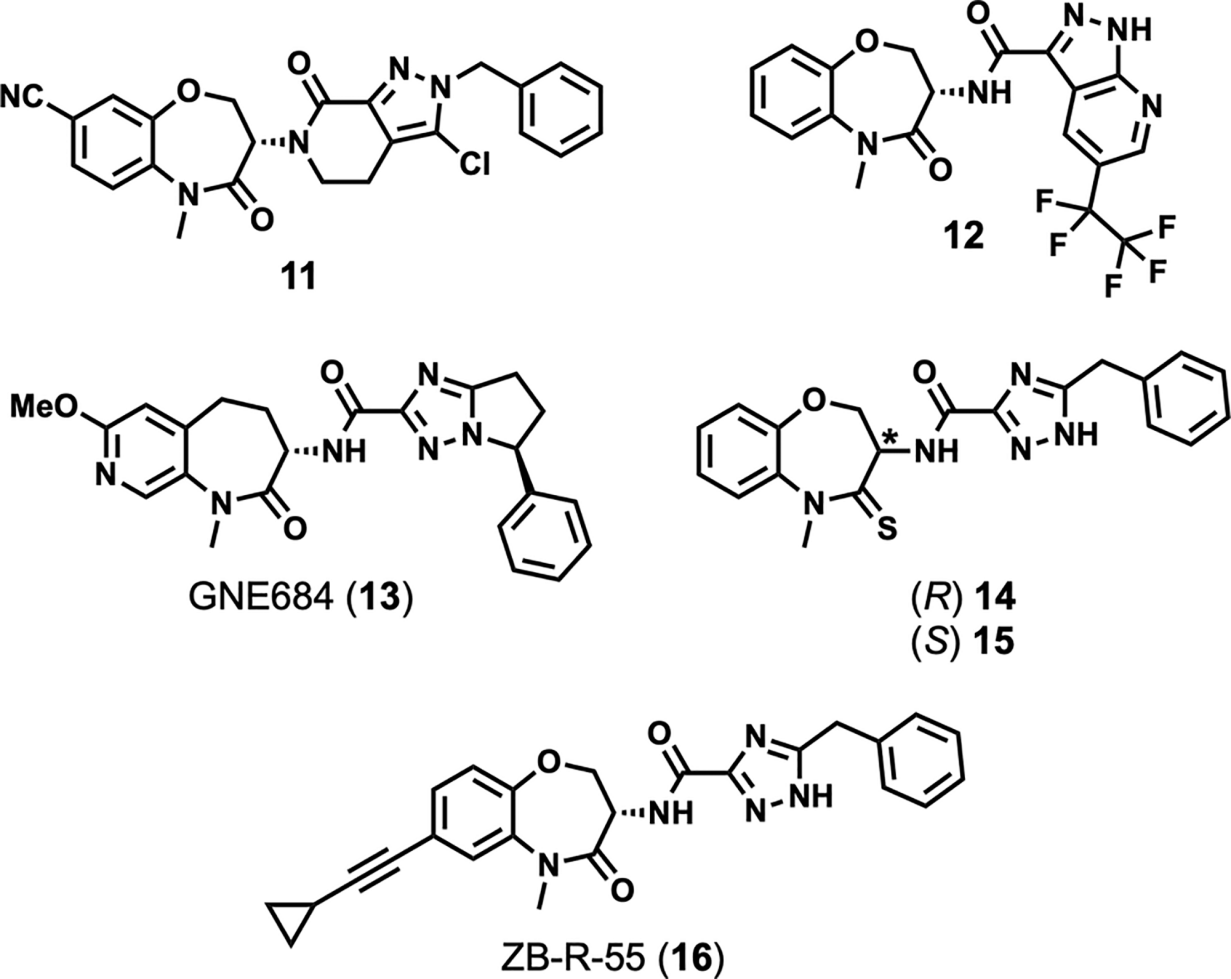
Other examples of the benzoxazepinone class.

Following the disclosure of GSK′481 and its X-ray structure, other teams also began working on the benzoxazepinone scaffold. Takeda developed benzoxazepinone **11** (Figure 6) through the hybridization of a HTS hit and GSK′772 and subsequent optimization with the aid of structure-based drug design.^124^ Benzoxazepinone **11** showed the best balance of potency (*K*_i_ = 0.91 nM), RIPK1 residence time (*t*_1/2_ = 210 min by TR-FRET assay), microsomal stability (CL < 1 μL min^–1^ mg^–1^ in mouse and human models), and P-gp-mediated efflux (ER = 0.7).^124^ When tested against a panel of 406 kinases, **11** displayed high selectivity for RIPK1 at 10 μM (*S*_(50)_ = 0.00(2), *S*_(20)_ = 0.03), with the exception of LIMK2 (85% inhibition @ 10 μM).^124^ When tested against the Eurofins Panlabs panel of 106 other targets, **11** showed significant inhibition (>50% @ 10 μM) of only monoamine oxidase MAO-B (53%) and cannabinoid receptor CB2 (54%).^124^ Additionally, **11** exhibited high plasma exposure (AUC = 658 ng h mL^–1^), moderate plasma stability (MRT = 3.1 h), a brain exposure equivalent to 50 nM, and a brain-to-plasma ratio of 0.3 in mice.^124^ Moreover, **11** attenuated disease progression in a mouse EAE model of multiple sclerosis, making it a good tool compound for evaluating the inhibition of RIPK1 in *in vivo* mouse models of neurodegenerative disease.^124^

Genentech developed benzoxazepinone **12** in a campaign to optimize the physicochemical properties of this inhibitor class (Figure 6).^125^ Benzoxazepinone **12** showed the best balance of affinity (*K*_i_^app^ = 6.0 nM by ADP^2^ FI), potency (IC_50_ = 54 nM in HT29 TNF/BV6/zVAD-fmk), and selectivity (*S*_(50)_ = 0.00, *S*_(20)_ = 0.01) against 219 other kinases at 10 μM.^125^ Furthermore, **12** also exhibited low clearance (7.1 mL min^–1^ kg^–1^), a moderate volume of distribution (2.8 L kg^–1^), a long half-life (*t*_1/2_ = 4.8 h), and excellent oral bioavailability (63%).^125^ Genentech also developed **13** (GNE684, Figure 6), which is reportedly the least primate-specific type III inhibitor of RIPK1 known (9- and 33-fold by *K*_i_^app^ vs mouse and rat models, respectively).^35^ GNE684 was shown to be a potent inhibitor of necroptotic cell death *in vitro* across multiple human and mouse cell lines and also maintained the high RIPK1 specificity of the benzoxazepinone class (*S*_(20)_ = 0.00) against a 221 kinase panel at 10 μM.^35^ Assessment of the PK profile (5 mg kg^–1^) revealed that GNE684 has high clearance (CL_p_ = 49.2 mL min^–1^ kg^–1^), a moderate volume of distribution (*V*_d_ = 1.84 L kg^–1^), and a short half-life (*t*_1/2_ = 0.53 h).^35^ Despite this profile, GNE684 provided protection in several *in vivo* models of inflammatory disease, including TNF-driven SIRS, colitis-induced by NEMO deficiency in IECs, and collagen antibody-induced arthritis.^35^ Altogether, GNE684 is proving to be a good tool compound for *in vivo* models of necroptotic disease. For example, its lack of efficacy in KRAS mutant pancreatic tumor models and a B16 melanoma model suggests that these diseases are not driven by necroptosis.^35^

A series of thio-substituted benzothiazoles was recently developed based on the observation that the benzoxazepinone carbonyl in GSK′772 did not form any interactions with the hinge region of RIPK1 (Figure 6).^126^ Interestingly, where GSK′772 showed >70-fold better activity with the (*S*)-enantiomer (3.6 nM vs 279 nM in HT-29 cells stimulated with TNF, a SMAC mimetic, and zVAD.fmk), this substitution reduced this difference in activity to less than 10-fold (2.8 nM for **14** vs 22.6 nM for **15** in HT-29 cells stimulated with TNF, a SMAC mimetic, and zVAD.fmk).^126^ To explain this dramatic reduction in activity between the enantiomers, the authors modeled the three-dimensional conformations of the enantiomers. These calculations suggest that the enantiomers of GSK′772 would occupy vastly different conformations, whereas the conformations of **14** and **15** would be quite similar. The authors propose that the increased bond length of the C=S bond relative to the C=O bond might increase the steric hindrance, resulting in reduced flexibility and restricted conformations.^126^ A preliminary SAR around this scaffold also suggests it could be a promising lead for further development.

By appending alkyne substituents to GSK′772, another series of benzoxazepinones that occupy both the allosteric site and the ATP pocket was developed.^127^ Of the compounds prepared, the cyclopropyl analogue ZB-R-55 (**16**, Figure 6) was the most potent in cellular assays (IC_50_ = 0.34 nM against U937 cells stimulated with TNF, a SMAC mimetic, and zVAD.fmk) and enzymatic assays (IC_50_ = 5.7 nM by ADP-Glo and 16 nM by ^33^P-radiolabeled assay).^127^ ZB-R-55 retained the exquisite kinase selectivity of the benzoxazepinone class, showing <30% inhibition of kinase activity against the Reaction Biology Corp and Eurofins panels when tested at 1 μM.^127^ The pharmacokinetic profile was favorable in mice (3 mg kg^–1^ p.o. and 1 mg kg^–1^ i.v.), with high oral exposure (AUC_last_ = 15 018 ng h mL^–1^), a high volume of distribution (*V*_ss_obs_ = 902 mL kg^–1^), low clearance (CL = 3.54 mL min^–1^ kg^–1^) and excellent oral bioavailability (*F* = 99%).^127^ ZB-R-55 was then evaluated in the SIRS and LPS-induced models of lethal shock and sepsis, respectively.^127^ When tested against GSK′772 at the same dose (10 mg/kg), ZB-R-55 exhibited improved protection against hypothermia and improved survival (100% vs 80%).^127^ These data suggest ZB-R-55 is a promising candidate for further development.

#### 4.1.4 Other RIPK1 Inhibitors

As the kinase activity of RIPK1 is required for the induction of death receptor-mediated necroptosis, it is unsurprising that numerous efforts have been made to assess the ability of approved kinase inhibitors to block necroptosis through the inhibition of RIPK1. Such endeavors have identified ponatinib and pazopanib,^128^ sorafenib,^129^ and tozasertib^130^ as type I and II kinase inhibitors of RIPK1. While efforts have been made to generate more selective derivatives from these starting points, no useful tool compounds or clinical candidates have emerged.^131,132^ Similarly, other type II inhibitors of RIPK1 have been identified from targeted chemical library screens^133−136^ or from compounds where off-target activity was observed toward RIPK1 when developing inhibitors for other kinases.^137^ The key advantage of type II RIPK1 inhibitors is their ability to overcome the primate selectivity observed in all type III scaffolds;^118,133^ however, this advantage has so far been overshadowed by their poor kinome selectivity and suboptimal physicochemical properties. Additionally, efforts to optimize type I and type II RIPK1 inhibitors are hampered by the current lack of X-ray cocrystal structures of RIPK1 bound to these compounds. All the current structures are for the binding of type III inhibitors, which stabilize the inactive form of RIPK1. While type II inhibitors may bind to a similar RIPK1 conformation (although there is no experimental data to confirm this), type I inhibitors stabilize kinases in their active forms. We would predict the active conformation of RIPK1 to be quite different from the solved structures and to more closely resemble the active structure of PKA (Figure 3A), with the activation loop helix not structured, the αC helix swung inward with the formation of the classic salt bridge interaction between the αC helix and the VAIK motif, the L of the DLG motif oriented inward (resembling the canonical DFG-in conformation), and the regulatory spine intact. Consequently, no type I or type II RIPK1 inhibitor has entered the clinic or provided a tool for delineating necroptosis biology.

Denali Therapeutics has also developed several clinical candidates from their RIPK1 inhibitor program. Their first candidate, DNL104, was developed for treatment of ALS and Alzheimer’s disease.^138^ In a phase I ascending dose study (NTR6257), DNL104 exhibited marked inhibition of RIPK1 phosphorylation and demonstrated CNS-penetrant properties; however, its development was discontinued due to liver abnormalities observed in the multiple ascending dose cohort.^138^ Two molecules from the same company, DNL747 and DNL758, have also undergone phase Ia (NCT03757351 and NCT03757325) and phase Ib (NCT04469621) trials.^139^ More recently, Eli Lilly purchased the rights to Rigel Pharmaceuticals’ RIPK1 inhibitor R552, which has completed a phase I clinical trial and is planned to undergo phase II studies in autoimmune and inflammatory diseases. Additionally, GenFleet Therapeutics is recruiting for a phase I trial (NCT04676711) of their RIPK1 inhibitor GFH312. For more details on the patent literature describing RIPK1 inhibitors, the reader is directed to a recent review on this topic, which details the development of several compound classes discussed in this review as well as other emerging scaffolds that fall outside the scope of this work due to the lack of published data.^140^

**Figure 7:**
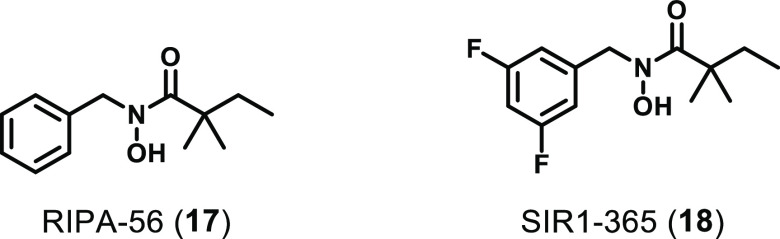
Chemical structures of other RIPK1 inhibitors.

RIPA-56 (**17**) was reported to be a highly potent, selective, and metabolically stable drug candidate for RIPK1-mediated disease (Figure 7).^141^ RIPA-56 was developed through *N*-hydroxylation of a hit identified through HTS of a 200 000 compound library against HT29 cells treated with a necroptotic stimulus (TNF, a SMAC mimetic, and zVAD.fmk).^141^ The authors report that RIPA-56 is potent against both human and murine cells *in vitro* (EC_50_ = 28 and 27 nM against HT29 and L929 cells, respectively), highly selective for RIPK1 over other kinases, metabolically stable *in vitro* (*t*_1/2_ = 128 and 35.5 min and Cl_int_ = 5.40 and 19.5 μL min^–1^ mg^–1^ in HLM and MLM, respectively), and efficacious in protecting mice from TNF-induced lethal shock and organ damage.^141^ However, upon *in vivo* pharmacokinetic (PK) testing (2 mg kg^–1^ i.v.), RIPA-56 also displayed low exposure (AUC_0–∞_ = 0.32 μg h mL^–1^), high clearance (Cl_p_ = 103 mL min^–1^ kg^–1^), and a large volume of distribution (*V*_ss_ = 27.8 L/kg).^141^ The authors also propose that RIPA-56 binds to the same allosteric pocket as other type III inhibitors of RIPK1. Furthermore, the fluorinated analogue SIR1-365 (**18**) was assessed for safety and efficacy in patients with severe COVID-19 (NCT04622332).

Various other inhibitors of RIPK1 have been identified (structures not shown); however, as many of these compounds were developed as inhibitors of other kinases or are hits from library screens without optimization, these scaffolds primarily serve as a demonstration of the increasing efforts being directed toward the discovery and development of RIPK1 inhibitors as therapeutics. The reader is directed to another review for further details on these recently identified scaffolds.^142^

### 4.2 Inhibitors of RIPK3

RIPK3 presents an attractive target for the inhibition of necroptosis, as it is downstream of RIPK1 and can potentially circumvent the issues associated with RIPK1’s other cellular functions. Furthermore, RIPK3 directly phosphorylates MLKL, so inhibiting its catalytic activity, oligomerization, or interaction with MLKL could also present unique opportunities to inhibit necroptosis at a late stage of pathway engagement.

#### 4.2.1 GSK Compounds

**Figure 8:**
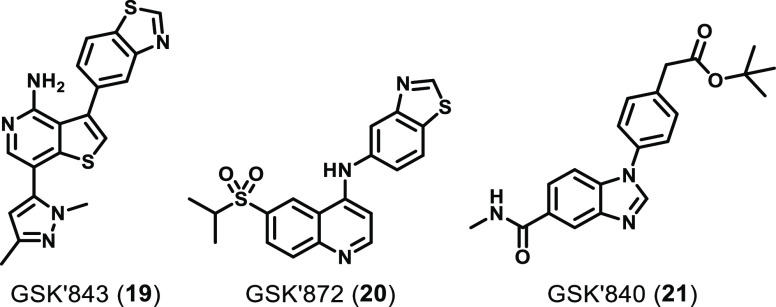
Chemical structures of RIPK3 inhibitors developed by GSK.

GSK first developed the RIPK3 inhibitors **19** (GSK′843) and **20** (GSK′872) (Figure 8) through HTS using a fluorescence polarization (FP) assay and subsequent optimization of the hits.^3^ These two compounds were shown to be potent in FP (IC_50_ = 8.6 and 1.8 nM, respectively) and ADP-Glo (IC_50_ = 6.5 and 1.3 nM, respectively) biochemical assays and upon necroptotic stimulation in both human (HT29 + TNF, a SMAC mimetic, and zVAD.fmk) and murine cells (3T3-SA + TNF and zVAD.fmk; or PEC, L929, SVEC and BMDM + TNF, a SMAC mimetic, and zVAD.fmk) *in vitro*.^3,68^ Furthermore, both compounds showed >1000-fold selectivity for RIPK3 over 291 other kinases at 1 μM (*S*_(20)_ = 0.24 and 0.14, *S*_(50)_ = 0.11 and 0.07, respectively) and did not inhibit RIPK1 activity, although data for the RIPK1 assays were not published.^68^ GSK′843 and GSK′872 inhibited RIPK2 activity by 84% and 76%, respectively, when assayed at 1 μM.^68^ This RIPK2 inhibitory activity must be taken into consideration when employing these compounds to investigate necroptosis in the context of autoinflammatory diseases.

**Figure 9:**
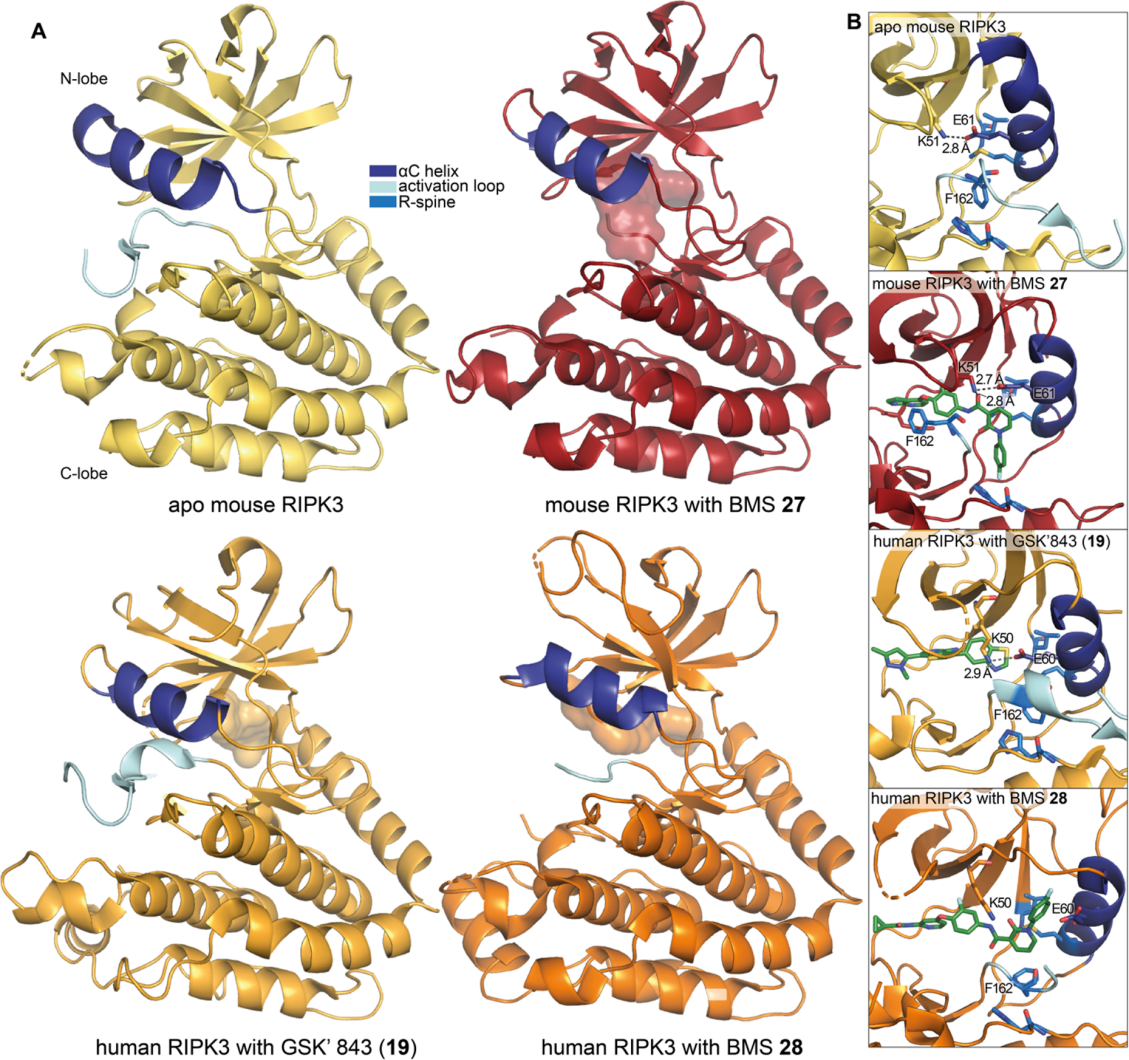
Selected crystal structures of RIPK3 with small-molecule inhibitors. (A) Apo mouse RIPK3 (yellow; PDB 4M66)^144^ compared to BMS **27**-bound mouse RIPK3 (red; PDB 6OKO),^145^ human RIPK3 bound to GSK′843 (**19**, light orange; PDB 7MX3),^143^ and BMS **28** (orange; PDB 7MON);^143^ the compounds are shown in the surface view. (B) A comparison between the active sites of all published RIPK3 structures, highlighting the repositioning of the regulatory spine and DFG motif residue F162. Compounds are shown in stick format.

Recently, GSK′843 was used as a tool compound to enable the determination of the first human RIPK3 crystal structure (PDB 7MX3).^143^ The compound binds in the expected type I inhibitor mode, and human RIPK3 is in the active conformation with the K50–E60 salt bridge intact and much of the activation loop resolved (Figure 9A). The protein conformation is similar to that of apo mouse RIPK3 (PDB 4M66).^144^

Later, GSK also identified the inhibitor **21** (GSK′840) through a DEL screen against the RIPK3 kinase domain (residues 2–328).^68^ GSK′840 was shown to bind and inhibit RIPK3 more potently than either GSK′843 or GSK′872 in biochemical assays (IC_50_ = 0.9 and 0.3 nM by FP and ADP-Glo, respectively), to be more potent in cellular necroptosis assays (HT29 + TNF, a SMAC mimetic, and zVAD.fmk; IC_50_ values not disclosed), and to be a more selective RIPK3 inhibitor when tested against a panel of 300 kinases at 1 μM (*S*_(20)_ = 0.07, *S*_(50)_ = 0.02).^68^ Interestingly, GSK′840 is inactive in murine cells, suggesting that it might bind to RIPK3 in a manner distinct from GSK′843 and GSK′872 that results in conformational changes that are unfavorable (or ineffective) in murine RIPK3. Unfortunately, no crystallographic data have been reported for GSK′840 to clarify this species specificity. It was observed that while these RIPK3 inhibitors inhibit RIPK1-independent necroptosis, in the absence of a caspase inhibitor they also induce RIPK3-dependent apoptosis at high concentrations (>1 μM).^68^ This has raised some toxicity concerns for the development of RIPK3 inhibitors as anti-inflammatory therapies and may explain why no RIPK3 inhibitor has yet entered the clinic.^60^

**Figure 10:**
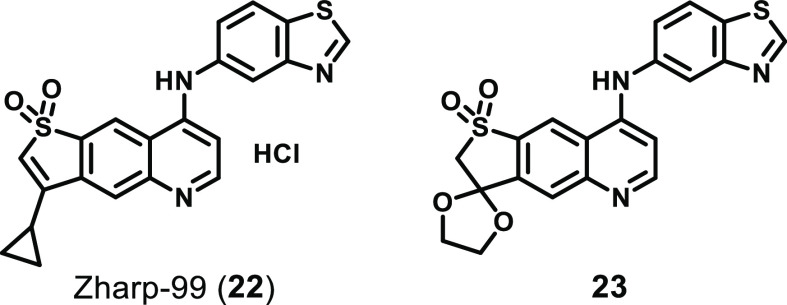
Recently developed cyclized analogues of GSK′872.

Recently, two cyclized analogues of GSK′872 have been reported, namely, Zharp-99 (**22**)^146^ and compound **23**(147) (Figure 10). Zharp-99 demonstrated improved efficacy when compared to GSK′872 in cell lines treated with necroptotic stimuli: human HT29 and mouse embryonic fibroblasts (MEF) stimulated with TNF, a SMAC mimetic, and zVAD.fmk; mouse and rat BMDMs stimulated with LPS and zVAD.fmk; and HSV-1-infected murine L929 cells.^146^ Zharp-99 was also shown to inhibit RIPK3 and MLKL phosphorylation in both human HT29 and mouse L929 cells and to inhibit death arising from forced RIPK3 dimerization in NIH3T3 cells expressing FKBP-tagged mRIPK3.^146^ However, Zharp-99 was unable to inhibit the formation of the RIPK1–RIPK3 necrosome complex in HT-29 cells stably expressing Flag–RIPK3 or MLKL polymerization in HeLa cells expressing a construct encoding an inducibly dimerizable form of the killer N-terminal region of MLKL.^146^ Furthermore, Zharp-99 is able to directly bind RIPK3 (*K*_D_ = 1.35 nM) and block its kinase activity (IC_50_ < 1 μM by ADP-Glo) and has no impact on RIPK1 kinase activity at 10 μM; however, its wider kinome selectivity has not yet been determined.^146^ The toxicity profile of Zharp-99 was also evaluated, where it showed minimal (<10%) inhibition of cytochrome P450 isozymes (CYP3A4, CYP2D6, CYP1A2, CYP2C9, and CYP2C19) at 10 μM and low inhibition of hERG (IC_50_ > 10 μM).^146^ However, Zharp-99 was found to induce on-target apoptosis to a greater extent than GSK′872 in MEF cells, suggesting that this molecule is more relevant as a tool compound than as a lead for clinical development.^146^ Zharp-99 also demonstrated moderate clearance (Cl_int_ = 212 and 67 mL^–1^ min^–1^ kg in MLM and RLM, respectively) and low stability (*t*_1/2_ = 26 and 36 min in MLM and RLM, respectively) *in vitro*, which translated into good exposure (AUC = 8.2 μg h mL^–1^ at 10 mg kg^–1^ dosed p.o.), a moderate clearance (33 mL min^–1^ kg^–1^) and volume of distribution (4.4 L/kg), and a short half-life (*t*_1/2_ = 1.5 h) *in vivo*.^146^ Importantly, administration of Zharp-99 (5 mg/kg, i.p.) also provided strong protection against lethal shock and hypothermia in a TNF-induced *in vivo* SIRS model.^146^

Compared to GSK′872, **23** (Figure 10) demonstrated improved cellular potency in both human HT29 and murine MEF cells (IC_50_ = 0.42 and 0.54 μM vs 1.51 and 2.51 μM, respectively) and no toxicity at 20 μM.^147^ While **23** was shown to be 1000-fold more selective for RIPK3 over RIPK1 in a direct-binding assay (*K*_D_ = 7.1 and 7200 nM, respectively), the broader aspects of its selectivity have not been fully explored, with the inhibition of only seven other kinases being investigated.^147^ Furthermore, **23** displayed moderate inhibition of CYP1A2 and 2C19 (37% and 20% at 10 μM, respectively) and minimal inhibition of hERG (IC_50_ > 30 μM).^147^ The DMPK profile of **23** also revealed high levels of plasma protein binding in human, rat, and mouse models (>97% in all cases), a low efflux ratio (0.72), and poor metabolic stability in human and mouse liver microsomes, with short half-lives (*t*_1/2_ = 28.75 and 20.26 h, respectively) and high clearance (Cl_int_ = 60 and 270 mL min^–1^ kg^–1^, respectively).^147^ Interestingly, the ketal functionality present in **23** was shown to be stable in simulated gastric fluid (pH 1.2) over 24 h, and administration of **23** (5 mg/kg, i.p.) also provided strong protection against lethal shock and hypothermia in a TNF-induced *in vivo* SIRS model.^147^

#### 4.2.2 Aminobenzothiazole Compounds

**Figure 11:**
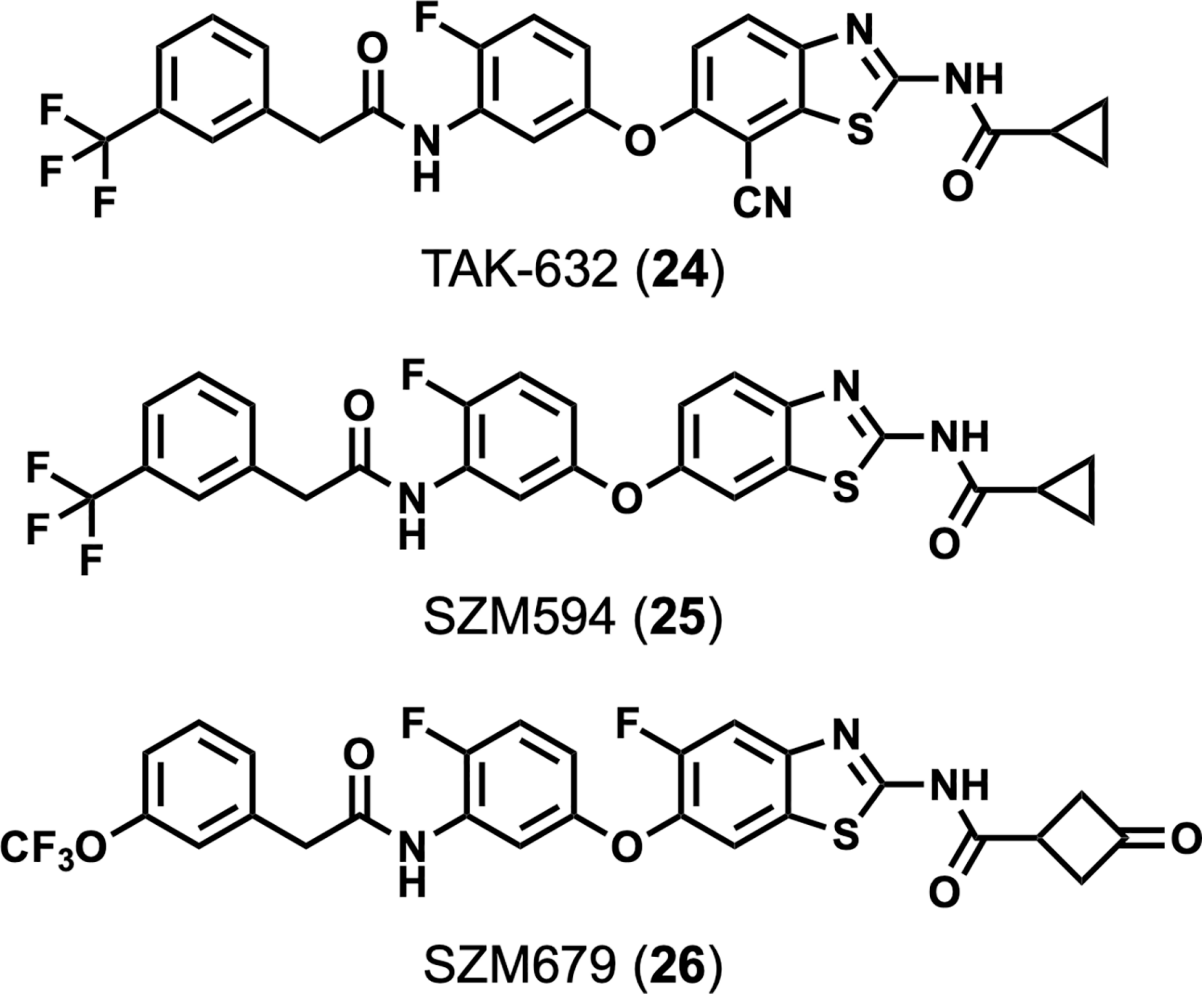
Chemical structures of aminobenzothiazole-based inhibitors.

A screen of 500 fluorinated compounds in an MTT assay against human HT29 cells treated with a necroptotic stimulus (TNF, a SMAC mimetic, and zVAD.fmk) identified aminobenzothiazole **24** (TAK-632) as a novel scaffold for RIPK3 inhibitors (Figure 11).^148^ TAK-632 was able to inhibit necroptosis in human THP-1 and U937 cells (treated with TNF, a SMAC mimetic, and zVAD.fmk), murine L929 and J774A.1 cells (treated with TNF and zVAD.fmk), and HT29 cells treated with Fas in addition to RIPK1-independent necroptosis induced by Poly(I:C) plus zVAD.fmk in RIPK1-deficient MEF cells.^148^ Furthermore, TAK-632 was shown to inhibit RIPK3 kinase activity in the KINOMEscan and [γ-^32^P] ATP radiometric assays (*K*_D_ = 326 nM and IC_50_ = 90 nM, respectively).^148^ However, TAK-632 was originally developed as a pan-RAF inhibitor and shows potent inhibition of not only RAF kinases (IC_50_ = 8.3 and 1.4 nM against B-RAF and C-RAF, respectively)^149^ but also RIPK1 (*K*_D_ = 105 and 480 nM by ADP-Glo and KINOMEscan, respectively) and the wider kinome (*S*_(35)_ = 0.14 against 90 kinases at 1 μM).^148^ Subsequent interrogation of the SAR around TAK-632 culminated in the development of benzothiazole **25** (SZM594, Figure 11).^148,150^ Compared to TAK-632, **25** was shown to be more potent in the HT29 cellular necroptosis assay (EC_50_ = 0.44 μM vs 1.44 μM), displayed reduced cytotoxicity (CC_50_ > 50 μM vs 36.5 μM), had greater affinity for RIPK3 (*K*_D_ = 81 nM) and selectivity over RIPK1 (*K*_D_ > 5,000 nM) in the KINOMEscan assay, and conferred better protection in the *in vivo* TNF and zVAD.fmk-induced SIRS mouse model (100% at 25 mg kg^–1^ vs 20% at 25 mg kg^–1^).^150^ Benzothiazole **25** still maintains significant activity against B-RAF (96.4% inhibition at 1 μM) and other kinases.^150^ While B-RAF is not involved in necroptosis, its inhibition might still complicate the interpretation of experimental results, especially *in vivo*. Further development of this scaffold delivered SZM679 (**26**), which is selective for RIPK1 over RIPK3 (*K*_D_ = 8.6 nM and >5 μM, respectively, by KINOMEscan; selectivity over other kinases not reported).^151^

#### 4.2.3 BMS Compounds

**Figure 12:**
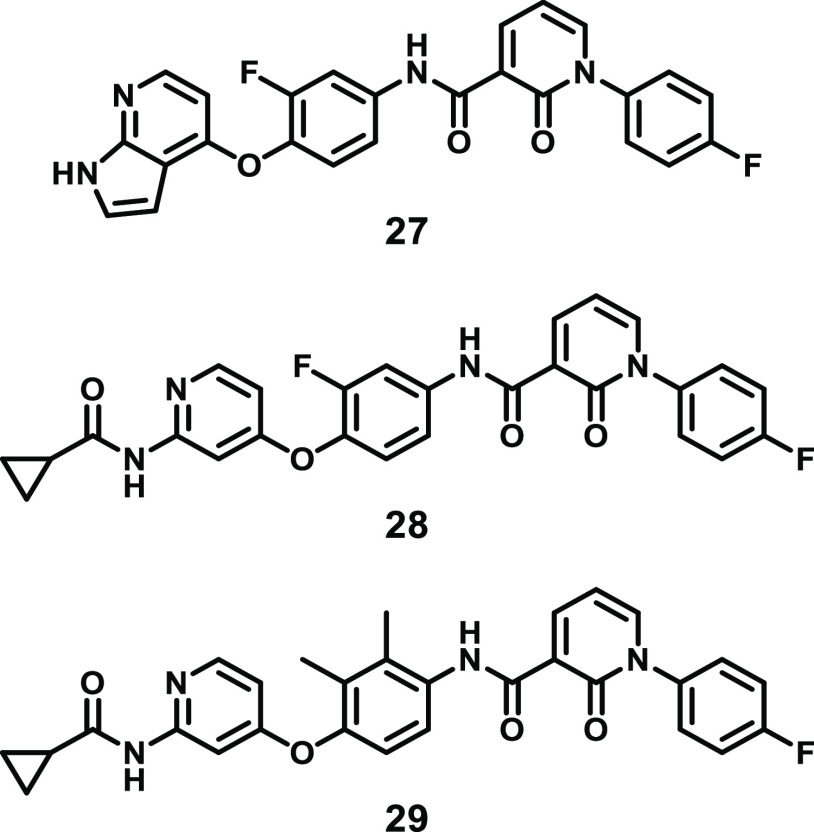
Chemical structures of RIPK3 inhibitors developed by BMS.

BMS undertook a HTS campaign with a homogeneous time-resolved fluorescence (HTRF) assay, followed by time-dependent inhibition studies, to specifically identify type II inhibitors of RIPK3.^145^ A search of the BMS internal library for compounds that were structurally related to the type II inhibitors identified in the HTS campaign then produced pyrrolopyridine **27** (Figure 12). Pyrrolopyridine **27** was shown to be selective for RIPK3 over RIPK2 and RIPK1 (IC_50_ = 0.18, >15, and 1.5 μM by HTRF, respectively); however, **27** was originally developed as an inhibitor of c-Met (IC_50_ = 5.7 and 1.9 nM by HTRF and [γ-^32^P] ATP radiometric assays, respectively) and also exhibits strong inhibition of FLT-3 and VEGFR-2 (IC_50_ = 2 and 27 nM, respectively, by [γ-^32^P] ATP radiometric assay).^145,152^

Importantly, pyrrolopyridine **27** enabled access to an inhibitor-bound mouse RIPK3 X-ray crystal structure (residues 1–313, C111A, C-terminal His_10_ tag; PDB 6OKO, Figure 9A).^145^ Compared to the apo mouse RIPK3 structure (PDB 4M66),^144^ the DFG motif has flipped into a classical DFG-out conformation, confirming that pyrrolopyridine **27** adopts the targeted type II binding mode (Figure 9B). The terminal aromatic group binds deeply into the hydrophobic allosteric site of RIPK3 and replaces the F162 of the DFG, exposing the D161 backbone and facilitating the formation of H-bond interactions with the pyridone oxygen. The positioning of this terminal aromatic group also stabilizes the R-spine, with residues H141, M65, and L76 remaining in similar positions compared to those in the apo structure. Interestingly, **27** makes a H-bond with K51 of the VAIK motif and stabilizes the catalytically essential E61–K51 interaction; however, the position of the DFG motif would still prevent kinase activity.

More recently, the closely related compound **28** (Figure 12) was used to determine the structure of the human MLKL pseudokinase and human RIPK3 kinase domain complex (PDB 7MON, Figure 9A).^143^ Interestingly, unlike that in the mouse RIPK3 **27**-bound structure (PDB 6OKO),^145^ the R-spine of human RIPK3 remains intact upon compound binding due to the alternate positioning of the fluorophenyl tail group in the human structure. The tail group sits adjacent to the αC helix, which is pushed away from the center of the N-lobe so that the K50–E60 salt bridge cannot form (side chains separated by 7.4 Å, Figure 9B). Therefore, the kinase is in an inactive conformation even though F161 of the DFG motif is not flipped out as it is in the inactive mouse RIPK3 structure bound to compound **27**. Despite the differences in the DFG motif conformation and the presence or absence of the K–E salt bridge in the inactive human and mouse RIPK3 structures (PDB 7MON and 6OKO, respectively) both show a translated αC helix and a marked shortening of the resolved section of their activation loop in comparison with their active conformation counterpart (PDB 7MX3 and 4M66, respectively; Figure 9A).

Through comparison of the crystal structures of **27** bound to mouse RIPK3 and c-MET kinase (PDB 3CE3), the structure-based optimization of this scaffold was performed, leading to the development of aminopyridine **29** (Figure 12).^145^ Aminopyridine **29** is a more potent inhibitor of RIPK3 (IC_50_ = 9.1 nM) with improved selectivity over RIPK1 and c-MET (IC_50_ = 5.5 and 1.1 μM, respectively). However, screening against an undisclosed number of other kinases revealed that **29** inhibits 42 other kinases with IC_50_ values of <1 μM, potentially limiting its utility as a tool to interrogate RIPK3 biology.^145^ Furthermore, the anti-necroptotic activity of these compounds has not yet been demonstrated either *in vitro* or *in vivo*.

#### 4.2.4 Other RIPK3 Inhibitors

Numerous efforts have been pursued to investigate approved kinase inhibitors in the context of inhibiting RIPK3. These studies have identified ponatinib,^128^ dabrafenib, regorafenib, vemurafenib, and sorafenib as inhibitors of RIPK3.^153^ However, these inhibitors display a lack of kinome selectivity. This is highlighted by the fact that both ponatinib and sorafenib are also inhibitors of RIPK1,^128,129^ limiting their development as therapies for necroptotic diseases.

**Figure 13:**
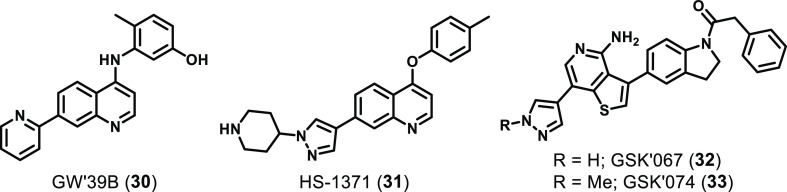
Chemical structures of other identified RIPK3 inhibitors.

HTS efforts have also identified other putative type I and II RIPK3 inhibitor scaffolds^154−156^ (Figure 13). The quinoline GW′39B (**30**) was identified by screening against NIH-3T3 cells expressing a RIPK3 construct that kills cells following inducible dimerization.^154^ GW′39B was able to inhibit RIPK3 in this assay (EC_50_ = 73.6 nM), protect both human and murine cells from diverse necroptotic stimuli, and inhibit MLKL phosphorylation and oligomerization.^154^ However, because GW′39B was originally developed as a RET kinase inhibitor,^157^ its kinase specificity and potential to activate RIPK3-dependent apoptosis remain of outstanding interest and preclude its use as a tool compound at this stage. HS-1371 (**31**), another quinoline, was also identified as a potent RIPK3 inhibitor (EC_50_ = 20.8 nM) in another HTS of known kinase inhibitor chemotypes.^155^ While HS-1371 inhibited RIPK3 autophosphorylation and rescued both human and murine cell lines from various necroptotic stimuli *in vitro*, it also showed apoptosis-related cytotoxicity similar to GSK′872.^155^ Additionally, the kinase specificity of HS-1371 has yet to be evaluated, which is an important consideration due to its original development as an ALK inhibitor.^158^ Two thienopyridines, **32** (GSK′067) and **33** (GSK′074, Figure 13), were identified by screening known kinase inhibitors against the MOVAS murine cell line treated with a necroptotic stimulus (TNF and zVAD.fmk).^156^ Despite the identified compounds being potent RIPK3 inhibitors in both human and murine cells *in vitro* and not driving RIPK3-mediated apoptosis, they are extremely nonspecific (GSK′074 *S*_(35)_ = 0.05 against 403 kinases at 100 nM) and have higher affinities for RIPK1 than RIPK3.^156^

### 4.3 Small Molecules Targeting MLKL

Because MLKL was only identified as the terminal effector of necroptosis as recently as 2012, the field of small-molecule targeting of MLKL is still in its infancy. Being a pseudokinase and therefore lacking any catalytic activity, the impact of targeting the pseudoactive ATP-binding site with ligands remains unclear. Fortunately, the development of small molecules that target other pseudokinase domains (*e.g.*, TYK2^159^ and JAK2^160^) suggests this could be a viable strategy.

#### 4.3.1 Necrosulfonamide

**Figure 14:**
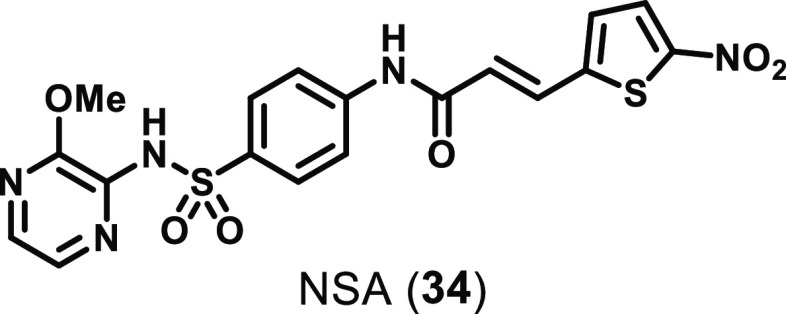
Chemical structure of the MLKL inhibitor necrosulfonamide (NSA).

The initial discovery of MLKL binders was somewhat serendipitous. In a screening campaign of a 200,000 compound library against HT29 cells treated with a necroptotic stimulus (TNF, a SMAC mimetic, and zVAD.fmk), a hit was identified that inhibited necroptosis with IC_50_ < 1 μM.^54^ Through investigation of the SAR around this hit, the optimized necrosulfonamide (NSA, **34**) was developed (Figure 14).^161^ NSA inhibited necroptosis in HT29 cells treated with TNF, a SMAC mimetic, and zVAD.fmk (IC_50_ = 124 nM); however, it had no effect in murine L929 or 3T3 cells treated with a necroptotic stimulus.^54^ The molecular target of NSA was determined to be downstream of RIPK3 and was identified as MLKL.^54^ NSA covalently binds to C86 of human MLKL; however, this C86 is a tryptophan in mouse MLKL, which explains the species selectivity of NSA.^54^

Recently, studies employing NMR experiments have indicated that upon covalent binding to human MLKL, NSA only forms weak interactions with the helical bundle and the first brace helix.^162^ This supports earlier molecular dynamics simulations suggesting that NSA forms a critical π–cation interaction with K157 of the second brace helix, leading to a reduction in the α-helical content of the brace helices and the subsequent formation of several new interactions between the pseudokinase domain, the second brace helices, and the helical bundle.^163^ NSA is therefore proposed to lock MLKL in an inactive conformation wherein it inhibits the release of the killer 4HB domain and subsequent oligomerization and translocation to the membrane.^10^ Unfortunately, further development of NSA has been limited due to its narrow SAR and the moderate potency and selectivity associated with targeting a surface cysteine residue rather than a specific binding pocket. Therefore, it should only be used as a tool compound for *in vitro* research and strictly only in studies of primate cells. In addition, other studies have shown that NSA can also impact gasdermin D (GSDMD) processing, either directly or upstream via caspase 1, and therefore can inhibit pyroptosis, another form of inflammatory cell death.^164,165^ This cross-reactivity with other cysteine-containing proteins is perhaps not surprising, especially when NSA is used at high concentrations.

#### 4.3.2 Xanthine-Based Ligands

**Figure 15:**
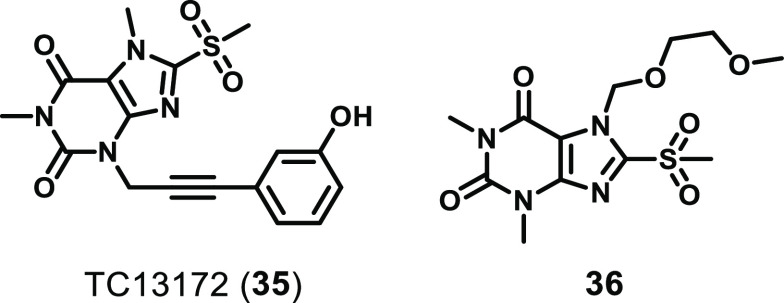
Chemical structures of xanthine-based MLKL ligands.

In a screening campaign of a 200 000 compound library against HT29 cells treated with a necroptotic stimulus (TNF, a SMAC mimetic, zVAD.fmk), a xanthine-based hit was identified that inhibited necroptosis with EC_50_ = 390 nM.^166^ This hit was then developed into TC13172 (**35**, Figure 15), which inhibited necroptosis in the same cellular assay with EC_50_ = 2.0 nM.^166^ Xanthine **35** was also shown to have no inhibitory effect on the kinase activity of RIPK1 or RIPK3 at 10 μM. Furthermore, **35** was shown to covalently bind to C86 of the MLKL 4HB, suggesting it has a similar mechanism of action to NSA. This was further demonstrated through the inability of **35** to inhibit the phosphorylation of MLKL by RIPK3 and its ability to prevent MLKL oligomerization and membrane translocation.^166^ Interestingly, 3D NOESY NMR experiments of **35** binding to the MLKL helical bundle (residues 2–154) suggest that the phenyl ring did not strongly interact with the helical bundle and was likely oriented toward the C-terminal end of the first brace helix.^162^ This suggested that this moiety might interact with the second brace helix or the pseudokinase domain in the context of full-length MLKL. Like NSA, **35** relies on the presence of C86 in human MLKL and primate orthologs and is therefore useful only as a tool for studies of MLKL in cell lines from primates.

**Figure 16:**
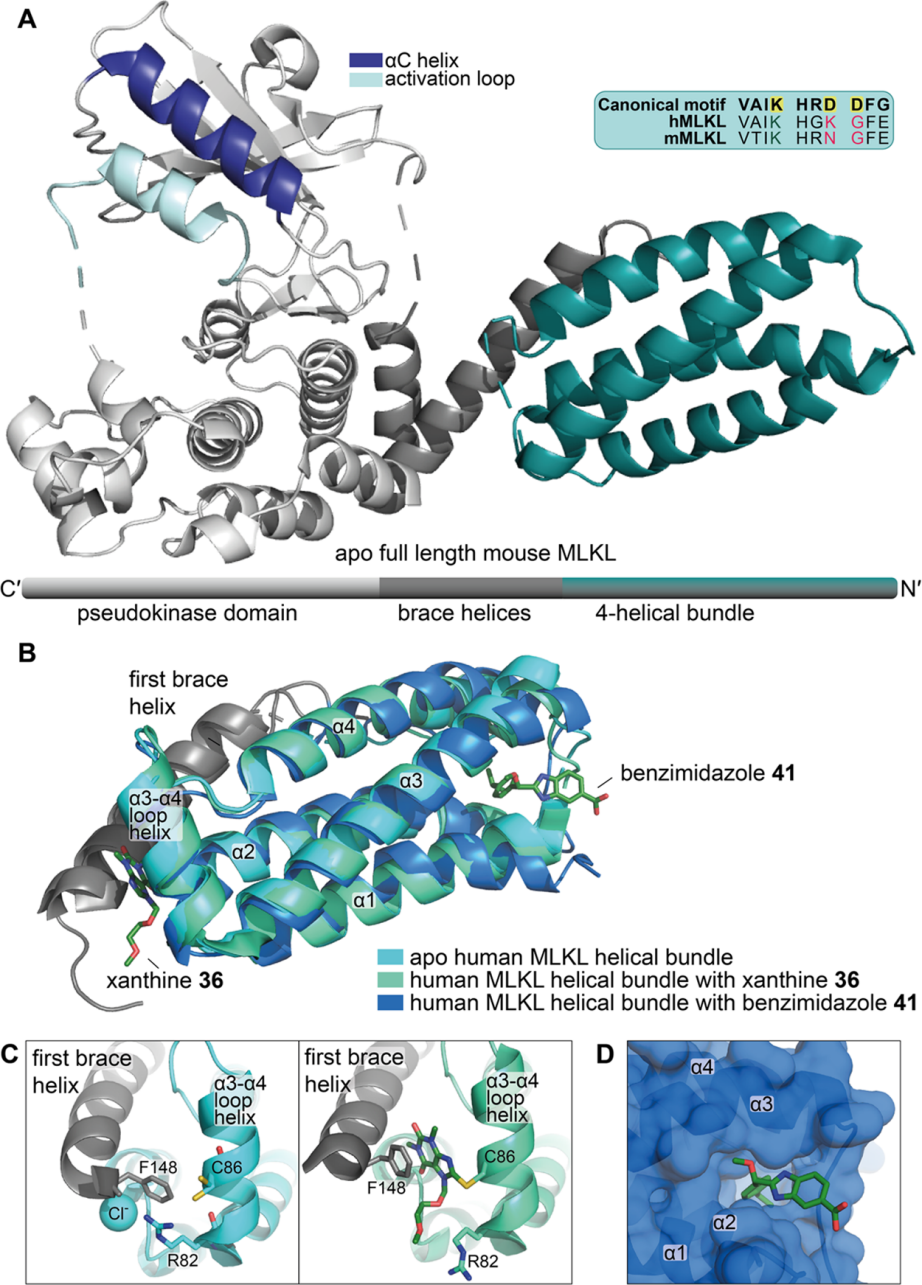
Selected crystal structures of MLKL with covalent small molecules interacting with the helical bundle domain. (A) Crystal structure of full-length mouse MLKL (PDB 4BTF),^6^ with the domain architecture highlighted (note: the domain architecture is drawn with the C-terminal to the left, *i.e.*, the reverse of the sequence direction, to correspond to the orientation of the structure). The inset shows that MLKL is catalytically inactive due to sequence divergences at key kinase catalytic motifs. (B) An overlay of apo (PDB 6ZVO),^162^ xanthine ligand-bound **36** (PDB 6ZZ1),^162^ and benzimidazole **41**-bound (PDB 7MN2)^167^ human MLKL helical bundle domain structures, with compounds labeled. (C) Comparison of apo and compound-bound structures at the compound binding site. (D) The binding pocket that benzimidazole **41** induces is shown in surface view. Compounds are shown in stick format.

To understand the mechanism of this class of xanthine-based compounds, Boehringer Ingelheim developed xanthine **36** and determined its impact on the structure of the MLKL N-terminal executioner domain.^162^ Using a combination of 3D NOESY NMR experiments (residues 2–154) and X-ray crystallography (residues 2–150, PDB 6ZZ1, Figure 16) the authors demonstrated that upon the covalent modification of C86 by **36**, the xanthine ring forms a π-stacking interaction with F148 of the first brace helix that stabilizes packing of the first brace helix against the helical bundle.^162^ Stabilization of this packing was proposed to lock the helical bundle and adjacent brace helices of MLKL in an inactive conformation, thereby impeding MLKL oligomerization and its necroptotic activity. The authors were also able to solve the apo structure (residues 2–150, PDB 6ZVO) and compare this to the xanthine-bound structure.^162^ This comparison revealed that the binding of xanthine **36** induces a rearrangement of only the side chains involved in or directly adjacent to compound binding (C86, F148, and R82). No impact on the overall architecture of the helical bundle was observed, and only minimal repositioning of the first brace helix upon compound binding was evident. Both the apo (PDB 6ZVO) and compound-bound (PDB 6ZZ1) structures closely resemble a previously described crystal structure of the human MLKL N-terminal domain cocrystallized with a monobody protein (PDB 6UX8).^12^ This monobody binds to an alternative site on the α4 helix but also inhibits MLKL translocation to the membrane and subsequent cell death.^12^

#### 4.3.3 Other Small Molecules Targeting the N-Terminal Domain of MLKL

**Figure 17:**
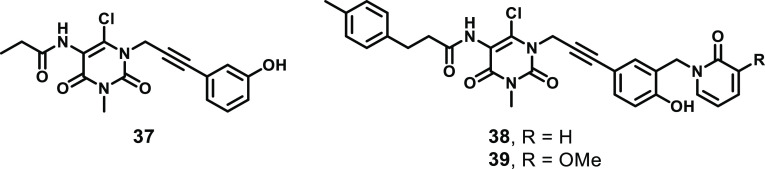
Chemical structures of uracil-based MLKL ligands.

A series of uracil compounds was recently developed (Figure 17) to overcome the liabilities of the xanthine-based small molecules targeting MLKL, namely, their high levels of reactivity toward nucleophiles under physiological conditions and the potential for off-target toxicity.^168^ A scaffold-morphing strategy identified the hit compound **37**, with EC_50_ = 3380 nM in HT29 cells treated with necroptotic stimulus (TNF, a SMAC mimetic, and zVAD.fmk). Elaboration of the SAR around this scaffold resulted in the development of uracils **38** and **39** (Figure 17) with markedly improved potencies (EC_50_ = 82 and 31 nM, respectively).^168^ Importantly, these optimized uracils showed little-to-no inhibition of RIPK1 (18 and 0% at 10 μM, respectively) and no inhibition of RIPK3 in ADP-Glo enzymatic assays, as well as no effects on the level of MLKL phosphorylation. Furthermore, no inhibition of cell proliferation or cell survival was observed when HT29 cells were treated with 5 μM **38** or **39**, and the reactive 6-chloro group showed reduced reactivity toward glutathione (GSH) compared to xanthine **35** (*t*_1/2_ = 48 or 160 h, respectively, compared to 20 min) when incubated together in DPBS buffer, suggesting a reduced propensity for off-target toxicity.^168^

It was observed that these uracils did not inhibit necroptosis in MEF cells (stimulated with TNF, a SMAC mimetic, and zVAD.fmk), suggesting that they targeted the Cys86 in human MLKL that is not present in mouse MLKL. Furthermore, the binding of both uracil **38** and **39** to MLKL was shown to outcompete that of TC13172 **35** in a pull-down assay.^168^ The binding of **38** to Cys86 was then confirmed by incubation with the MLKL protein and analysis via MS/MS, as well as mutagenesis studies wherein **38** and **39** failed to inhibit necroptosis in HT29 cells transfected to express a C86S mutant.^168^ Immunoblotting assays indicated that the uracils were partly able to reduce MLKL oligomerization, while immunofluorescence experiments showed that they inhibited the translocation of MLKL to the plasma membrane.^168^ However, without available structures of **38** or **39** bound to full-length MLKL or structures of MLKL oligomers, the authors can only speculate that these compounds disrupt an unidentified amino acid that has no impact on MLKL oligomerization but is integral for its membrane translocation.

**Figure 18:**
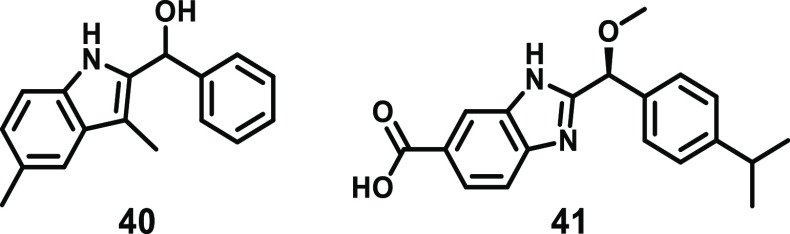
Chemical structures of indole **40** and optimized benzimidazole **41**.

Recently, a series of fragments that bind noncovalently to the MLKL executioner domain have been reported (Figure 18).^167^ Indole **40** was identified through an NMR-based fragment screening against the MLKL executioner domain (residues 2–154) and was shown to have a binding affinity (*K*_D_) of 933 μM by NMR titration.^167^ Optimization of this scaffold led to the development of benzimidazole **41** (Figure 18), with an improved *K*_D_ of 50 μM.^167^ Importantly, the switch from an indole to benzimidazole scaffold afforded increased chemical stability upon chiral separation, facilitating the determination of the (*S*)-enantiomer as the active species. Furthermore, alkylation of the chiral alcohol fills an induced pocket in the binding site, while the carboxylate group was appended to improve compound solubility.^167^

The NMR costructure of benzimidazole **41** bound to the MLKL executioner domain indicated that these compounds occupy an induced pocket near the N-terminus of the protein, between the pairs of α-helices that form the four-helix bundle (Figure 16D).^167^ The *iso*-propyl-phenyl group projects into the hydrophobic core of the four-helix bundle domain, with the carboxylate group oriented toward the solvent. Interestingly, the binding of **41** to the four-helical bundle domain does not markedly modify the structure compared to the unbound protein.^162^ Furthermore, the binding site of these compounds was shown to also be the binding site of the detergent monomer nonyl-maltoside, which when added with inositol phosphates can be used to induce the activation of the MLKL executioner domain *in vitro*.^169^ Due to the shared nature of this binding site, the authors postulate that competition to detergent binding is a potential mode of action for MLKL inhibition. Unfortunately, benzimidazole **41** was unable to demonstrate efficacy in any cellular assays due to poor membrane permeability (3.1 × 10^–8^ cm s^–1^ in a PAMPA assay) or in a liposome leakage assay, presumably because it was out-competed by detergents under the assay conditions as a result of its low binding affinity for MLKL (*K*_D_ = 50 μM).^167^ The further optimization of compounds to probe this newly identified binding site is of great interest, as it might illuminate possibilities to inhibit necroptosis at a late stage, such as MLKL multimerization or membrane interaction, and avoid the aforementioned issues of targeting the upstream kinases RIPK1 and RIPK3 or other regions of MLKL.

#### 4.3.4 Aminopyrimidines

**Figure 19:**
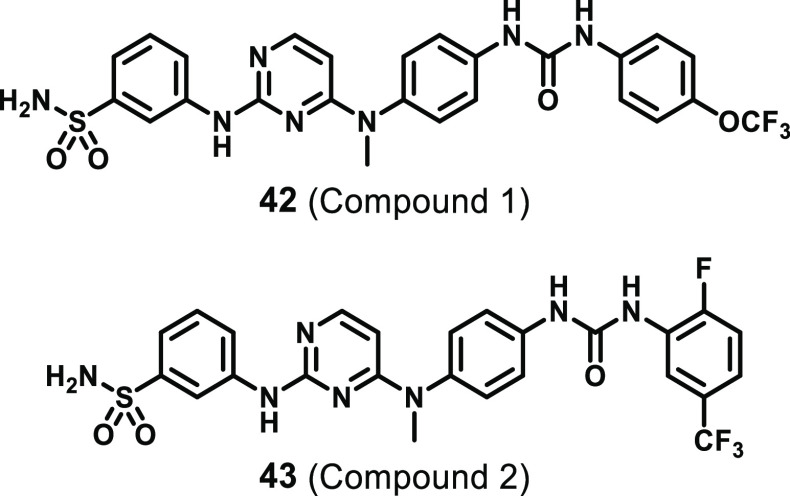
Chemical structures of aminopyrimidine-based MLKL ligands.

A screen of known kinase inhibitors against recombinant mouse MLKL using thermal shift assays identified aminopyrimidine **42** (compound 1, Figure 19) as a MLKL binder.^9^ It was demonstrated through surface plasmon resonance (SPR, *K*_D_ = 9.3 μM) and saturation transfer difference NMR (STD-NMR) studies that **39** bound to the nucleotide-binding site of MLKL. Furthermore, **42** was able to inhibit necroptosis in MDFs (IC_50_ < 50 nM, treated with TNF, a SMAC mimetic, and QVD-OPh) and retarded the translocation of MLKL to the membrane.^9^ The inhibitory properties of **42** against human MLKL were later examined, where it demonstrated a moderate affinity for MLKL (*K*_D_ = 530 nM), poor kinome selectivity (inhibited 56 out of 403 kinases at 1 μM) including the inhibition of RIPK1 and RIPK3 (*K*_D_ = 64 and 680 nM, respectively), moderate cellular potency in TNF-stimulated FADD-deficient Jurkat cells (EC_50_ = 1.85 μM), and toxicity at higher concentrations (∼2 μM).^170,171^ As **42** was developed as a VEGFR2 inhibitor (IC_50_ = 2 nM),^172^ this lack of selectivity for MLKL is unsurprising.

More recently, an analogous compound (**43**, Compound 2, Figure 19) was described as a more potent inhibitor of necroptosis.^171^ Compared to **42**, aminopyrimidine **43** showed a reduced affinity for MLKL and RIPK3 (*K*_D_ = 1800 and 1900 nM, respectively) and an improved affinity for RIPK1 (K_D_ = 19 nM) in a competitive binding assay despite also showing relatively poor selectivity over the wider kinome (*S*_(35)_ = 0.31).^171^ Furthermore, the binding of **43** to all three key necroptosis effector proteins was confirmed through cellular thermal shift assays (CETSA) in both human U937 and murine MDF cells and *in vitro* photoaffinity labeling experiments. Interestingly, aminopyridine **43** was shown to be a more potent inhibitor of necroptotic cell death in U937 and HT29 cells (treated with TNF, a SMAC mimetic, and QVD-OPh or IDN-6556) relative to **42**, with similar levels of toxicity.^171^ By employing a series of knockout cell lines, the authors also demonstrated that this cell death was mediated through caspase-dependent and BAX/BAK-independent apoptosis, which was also independent of RIPK1, RIPK3, and MLKL, suggesting that the cell death was mediated through the complex pharmacology of this scaffold.^171^ Finally, aminopyrimidine **43** was evaluated *in vivo* using a TNF-induced systemic inflammatory response syndrome (SIRS) model, where treatment corresponded to a modest delay (3 h) in hypothermia and a reduced incidence of death (one death in the treatment arm vs three deaths in the vehicle control).^171^

**Figure 20:**
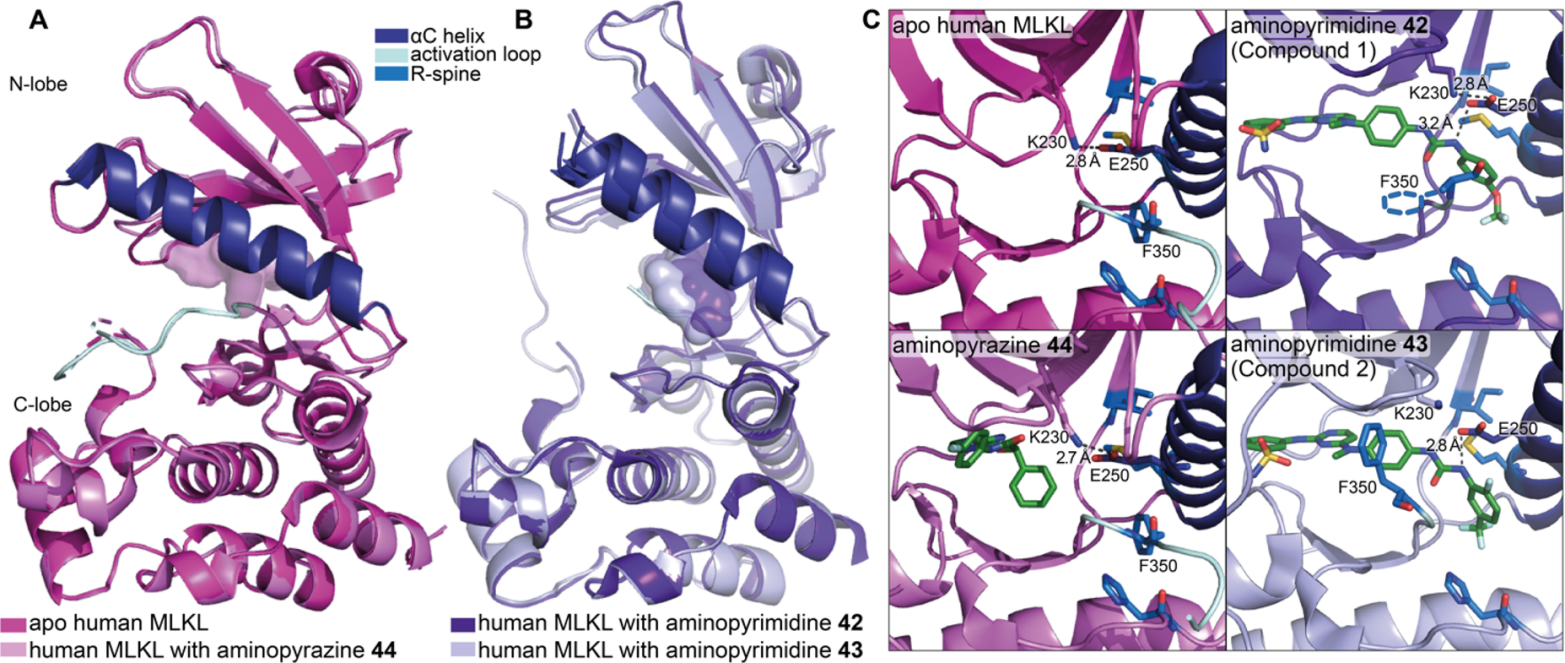
Selected crystal structures of the MLKL pseudokinase domain with and without small-molecule ligands. (A) Human MLKL bound to aminopyrazine **44** (PDB 5KO1)^170^ has a similar conformation to the apo human MLKL pseudokinase domain (PDB 4MWI).^173^ (B) Human MLKL bound to aminopyrimidine **42** (PDB 5KNJ)^170^ has a similar conformation to human MLKL bound to aminopyrimidine **43** (PDB 6O5Z)^171^ and differs only slightly from apo human MLKL (panel A). (C) Comparison of the MLKL pseudoactive site in the apo and compound-bound structures, highlighting repositioning of F350, from the GFE motif in aminopyrimidine **42**- and **43**-bound human MLKL. Compounds are shown in surface format (panels A and B) or in stick format (panel C).

X-ray crystal structures of the human MLKL pseudokinase domain bound to **42** (residues 191–471, E366A and K367A; PDB 5KNJ)^170^ and **43** (residues 190–471; PDB 6O5Z)^171^ confirmed that these small molecules occupy the ATP-binding site and extend into the allosteric pocket in a type II conformation (Figure 20). The aminopyrimidine motif forms H-bonds with the hinge region, while the urea interacts with E250 of the αC helix, as is typical of a type II binding conformation. Binding of the terminal aryl group in the allosteric pocket modestly displaces the αC helix relative to the apo structure (PDB 4MWI),^173^ however, the αC E250-K230 H-bond is maintained. The trifluoromethoxy group of **42** displaces F350 of the GFE motif and, although the side chain is truncated, F350 appears to become positioned in a conformation analogous to a classical DFG-out motif. Despite these conformational changes, however, **42** does not prevent MLKL phosphorylation and therefore does not prevent RIPK3 binding.

#### 4.3.5 Other Small Molecules Targeting MLKL

**Figure 21:**
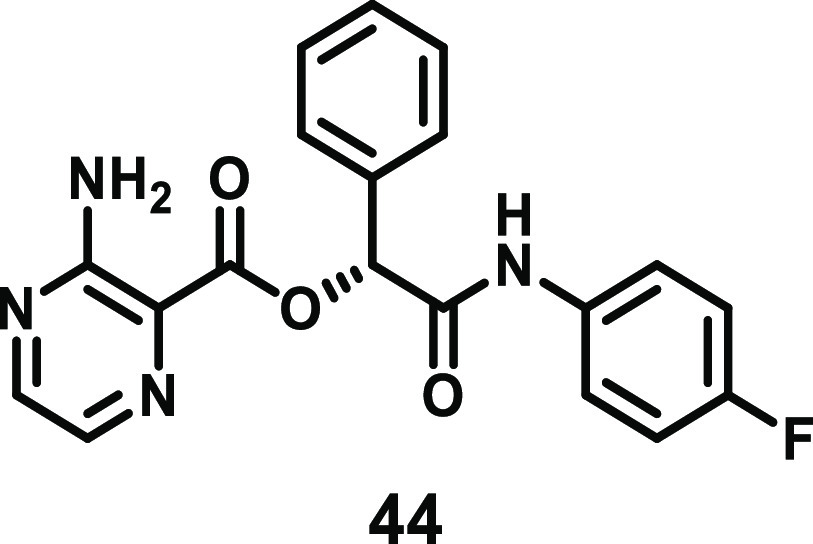
Chemical structure of aminopyrazine **44**.

Biogen screened a library of 5000 compounds using an ATP-competitive probe displacement assay to identify compounds that specifically bind the active site of MLKL but not RIPK1 or RIPK3.^170^ One hit this screen identified was crizotinib, a type I kinase inhibitor. Crizotinib had a higher affinity for MLKL than either RIPK1 or RIPK3 (*K*_D_ = 217, 1300, and 6700 nM, respectively) but showed no cellular activity against TNF-stimulated *FADD*^–/–^ Jurkat cells, even at 40 μM.^170^ Furthermore, crizotinib was developed as an inhibitor of c-MET and ALK (enzymatic IC_50_ < 1.0 nM) and inhibits other kinases with similar potency,^174^ making it unsuitable for specifically targeting MLKL in cellular or *in vivo* settings. This screen also identified aminopyrazine **44** (Figure 21), which was found to modestly bind MLKL (*K*_D_ = 230 nM) while being completely selective over RIPK1 and RIPK3 (*K*_D_ > 30 μM) and the broader kinome (inhibited only MLKL versus 403 kinases at 1 μM).^170^ Interestingly, the enantiomer had a markedly lower affinity for MLKL (*K*_D_ = 7.6 μM), suggesting it could potentially be a useful negative control. Unfortunately, aminopyrazine **44** showed no cellular activity against TNF-stimulated *FADD*^–/–^ Jurkat cells at 40 μM and was unable to inhibit MLKL phosphorylation by RIPK3.^170^

The X-ray crystal structure of aminopyrazine **44** bound to the MLKL pseudokinase domain (residues 191–471, E366A and K367A; PDB 5KO1) has also been obtained, which confirms that it occupies the ATP site in a type I binding mode (Figure 20A and C).^170^ The binding of **44** does not markedly change the conformation of MLKL compared to the apo structure (PDB 4MWI),^173^ except for the glycine-rich loop being raised slightly (3.9 Å at E213, Cα to Cα). However, this does not appear to translate to other conformational changes. As our understanding of the conformational changes that MLKL must undergo to be in an activated state are limited, one can only speculate that type I inhibitors (such as crizotinib and **44**) do not induce sufficient repositioning of key MLKL activity regulating elements (namely the αC helix or activation loop) to inhibit activity.^6,8,175^ The cocrystal structure of the mouse MLKL pseudokinase domain in complex with mouse RIPK3 (PDB 4M69)^144^ shows the repositioning of the MLKL αC helix to form an interface with mouse RIPK3 and a movement of the activation loop helix into the cleft between the N- and C-lobes. Interestingly, this conformation of the activation loop helix would induce minor clashes with both aminopyrazine **44** and aminopyrimidine **42**. A recently published structure of the human MLKL and RIPK3 complex (PDB 7MON) suggests that unlike in the mouse counterpart aminopyrazine **44** binding could be accommodated within the human MLKL RIPK3 complex.^143^ The binding of the type II inhibitor aminopyrimidine **42** would clash with F350 of the GFE (MLKL equivalent to DFG) motif, however, which is in the “in” conformation in the structure.^143^ Whether the complex could tolerate conversion to the “DFG out” conformation is unknown.

The claims and conclusions of the paper disclosing aminopyrazine **44** leave several outstanding questions. First, while the cellular assay described in this study did not show antinecroptotic activity, it remains of outstanding interest whether a more conventional HT29, L929, MDF, or MEF cell line treated with necroptotic stimuli would be impacted by the compounds studied. Further, the assertion that targeting the pseudokinase domain of MLKL has no functional impact on necroptosis is based on only a handful of compounds, none of which can be considered “optimized” inhibitors of MLKL. A larger number and greater diversity of compounds with more potent binding affinities need to be evaluated before such a conclusion can be made. More broadly, this conclusion definitively highlights the challenge associated with targeting pseudokinase pseudoactive sites because of their ancestral origins shared with paralogous kinases. MLKL functions as a protein switch, with the necroptotic function regulated by interconversion between an inactive “open” state and an active “closed” state.^6,7,9^ The authors’ own crystallographic studies demonstrate that aminopyrazine **44** binds to the closed state of MLKL, which is more suggestive that type I inhibitors do not stabilize a conformation of the pseudoactive site that inhibits the killer activity of the MLKL helical bundle. Additionally, the authors’ suggestion that aminopyrimidine **42**, a type II binder of MLKL, exerts its antinecroptotic activity primarily through inhibition of RIPK1 and not its binding to MLKL further weakens their claim. Overall, small molecule targeting MLKL with increased potency and selectivity are needed to better understand the effect that compounds engaging MLKL have on necroptotic signaling and to remove any confounding effects due to simultaneous RIPK1 or RIPK3 inhibition.

## 5 The Right Tool for the Job

Throughout this Perspective, we aimed to arm the reader with data-encompassing selectivity, affinity, PK/PD properties, and species specificity, which are critical for selecting the best tool compound for inhibiting necroptosis either *in vitro* or *in vivo*. Table 1 summarizes our suggestions for first-choice tool compounds, along with their limitations. Again, we remind the reader that for the purpose of this Perspective, when we refer to *in vivo* animal models of necroptotic disease, we are generally referring to the rodent models easily accessible to most researchers.

**Table 1:** Existing Tool Compounds and Their Applicability

compound	target protein	when to use	rationale	refs
GSK′772	RIPK1	√ cellular (primate) necroptosis assays	GSK′772 is highly potent and selective for RIPK1, with extensive characterization in PK/PD and preclinical models. It is unsuitable for use in nonprimate cells and *in vivo* models due to excessive primate selectivity	(120)
		√ biochemical assays		
		√ *in vitro* primary cell (primate) assays		
GNE684	RIPK1	√ cellular necroptosis assays	GNE684 is highly potent and selective for RIPK1, with similar levels of activity against primate and rodent orthologues. Due to some limitations with the PK/PD properties, GNE684 is more suitable for acute models of disease.	(35)
		√ biochemical assays		
		√ *in vitro* primary cell (primate) assays		
		√ *in vivo* models of necroptotic disease		
GSK′840	RIPK3	√ cellular necroptosis assays	GSK′840 is more potent and selective for RIPK3 than GSK′872 or GSK′843 but is only active against primate RIPK3.	(68)
		√ biochemical assays		
		√ *in vitro* primary cell (primate) assays		
GSK′872	RIPK3	√ cellular necroptosis assays	GSK′872 is active against both primate and rodent RIPK3. Caution must be taken when used *in vivo*, as toxicity can occur at concentrations >1 μM.	(3)
		√ biochemical assays		
		√ *in vitro* primary cell (primate) assays		
		√ *in vivo* models of necroptotic disease		
NSA	MLKL	√ cellular (primate) necroptosis assays	NSA can only bind and inhibit primate MLKL and not rodent orthologues. Its use should be limited to *in vitro* assays. It is important to note that additional studies have also linked NSA to pyroptosis.	(54, 164, 165)
		√ biochemical assays		
		√ *in vitro* primary cell (primate) assays		

## 6 Final Remarks

Continued efforts are still required to fully characterize the necroptosis pathway. For this, the ongoing development and correct use of chemical probes targeting RIPK1, RIPK3, and MLKL will be essential. Novel chemotypes and greater creativity are required to develop next-generation inhibitors of necroptosis that circumvent issues such as off-target activity, species specificity, and interference with the non-necroptosis functions of RIPK1 and RIPK3. Conversely, the potential of small-molecule activators of necroptosis remains unclear. These endeavors also require improved structural details of the necroptosis proteins, especially RIPK3 and MLKL, and their interactions.

Efforts to inhibit the necroptosis pathway have thus far focused on RIPK1, presumably because of its apical role and its implication in the pathway 16 years ago. Further research aimed at delineating the various kinase and scaffolding functions of RIPK1, as well as its roles in cell survival and apoptosis, will facilitate the further development of RIPK1 inhibitors as therapeutics for human diseases. An improved understanding of RIPK1 biology might provide further options for targeting RIPK1, such as altering its post-translational modifications other than the phosphorylation state (*e.g.*, ubiquitination), modulating the conformation of the kinase domain, disrupting protein–protein interactions, and possibly developing activators for certain applications.

The development of RIPK3 inhibitors has been hampered by a lack of publicly available crystallographic data to support structure-based design and the potential toxicity issues arising from the ability of RIPK3 inhibitors to induce RIPK3-dependent apoptosis. However, opportunities still exist for potentially targeting RIPK3. It might be possible to develop type III inhibitors that bind to the distal pocket created by the DFG-out conformation, as identified in the pyrrolopyridine **27**–mouse RIPK3 structure. While it is typically more difficult to target protein–protein interactions, especially without detailed structural knowledge of the necrosome, it might be possible to inhibit the interaction of RIPK3 with either RIPK1 or MLKL or indeed its own oligomerization.

Direct inhibition of MLKL represents an attractive option for generating antinecroptosis therapies because MLKL is the final effector of necroptosis, is expressed in a greater number of cell types than RIPK3, and might circumvent the issues observed with inhibition of RIPK1 (scaffolding function) and RIPK3 (activating apoptosis). Unfortunately, our understanding of the structural requirements for MLKL activation/inactivation is still lacking, as are our chemical tools to interrogate this. While type I binders of the pseudokinase domain might not be effective at reorganizing the αC helix and activation loop to prevent phosphorylation by RIPK3, allosteric binders (equivalent to type III kinase inhibitors) might have greater utility in this area, should they be developed. Such MLKL ligands might be able to inhibit release of the killer 4HB domain through noncovalent interactions, allowing their potential use *in vivo*. As our understanding of MLKL biology increases, it might also offer insight into ways we could design small molecules to target the oligomerization, translocation, or membrane interactions of MLKL.

## Abbreviations Used

BAK: BCL-2 homologous antagonist
BAX: BCL-2 associated X-protein
BMDM: bone marrow-derived macrophage
CB2: cannabinoid receptor type 2
CETSA: cellular thermal shift assay
CLint: intrinsic clearance
CNS: central nervous system
CRISPR: clustered regularly interspaced short palindromic repeats
Cas: CRISPR-associated protein
DAI: DNA-dependent activator of IFN regulatory factors
DAMPs: damage-associated molecular patterns
DPBS: Dulbecco’s phosphate-buffered saline
DEL: DNA-encoded library
DHP: dihydropyrazole
ds: doubled stranded
EAE: experimental autoimmune encephalomyelitis
ER: efflux ratio
ESCRT: endosomal sorting complexes required for transport
FADD: factor-associated suicide receptor-associated protein with death domain
FAS: factor-associated suicide receptor
FasL: factor-associated suicide receptor
FKBP: FK506 binding protein
FLT-3: fms-like tyrosine kinase
FP: fluorescence polarization
HB: helical bundle
GSDMD: gasdermin D
HLM: human liver microsome
hPXR: human pregnane X receptor
HTRF: homogeneous time-resolved fluorescence
cIAP1/2: cellular inhibitors of apoptosis 1 or 2
IDN: Idun
IEC: immune effector cell
IFN: interferon
IDO: indoleamine 2,3-dioxygenase
IFNAR1: interferon-α/β receptor subunit 1
IKK: inhibitor of nuclear factor κB kinase
IPMK: inositol polyphosphate multikinase
JAK2: janus kinase 2
KRAS: Kirsten rat sarcoma
LIMK2: LIM domain kinase 2
LPS: lipopolysaccharides
LUBAC: linear ubiquitin chain assembly complex
MEF: mouse embryonic fobroblast
MLKL: mixed lineage kinase domain-like protein
MLM: mouse liver microsomes
MRT: mean residence time
MAO-B: monoamine oxidase
NASH: nonalcoholic steatohepatitis
Nec: necrostatin
NF-κB: nuclear factor κB
NEMO: NF-κB essential modulator
NOD: nucleotide binding and oligomerization domain
NLRs: nucleotide binding and oligomerization domain-like receptors
NSA: necrosulfonamide
PAINS: pan-assay interference compounds
PDB: Protein Data Bank
PDOTS: patient-derived organotypic spheroids
PEC: parietal epithelial cells
poly(I:C): polyinosinic:polycytidylic acid
PsKD: pseudokinase domain
Rd10: retinal degeneration 10
RHIM: receptor-interacting protein homotypic interaction motif
RLM: rat liver microsome
RIPK1: receptor-interacting protein kinase 1
RIPK2: receptor-interacting serine/threonine kinase 2
RIPK3: receptor-interacting protein kinase 3
RP: retinitis pigmentosa
shRNA: short hairpin ribonucleic acid
siRNA: small interfering ribonucleic acid
SIRS: systemic inflammatory response syndrome
SMAC: second mitochondria-derived activator of caspases
STD: saturation transfer difference
TAK1: transforming growth factor-β-activated kinase 1
TAK: tat-associated kinase
TNF: tumor necrosis factor
TNFR1: tumor necrosis factor receptor 1
TLR3: toll-like receptors 3
TLR4: toll-like receptors 4
TRADD: tumor necrosis factor receptor-associated death domain
TRAF2: tumor necrosis factor receptor-associated factor 2
TR-FRET: time-resolved fluorescence energy transfer
TRIF: toll/interleukin-1 receptor/resistance protein-domain-containing adaptor-inducing interferon-β
ZBP1: Z-DNA binding protein 1
